# Protein-free domains in native and ferroptosis-driven oxidized cell membranes: a molecular dynamics study of biophysical properties and doxorubicin uptake

**DOI:** 10.3389/fmolb.2024.1494257

**Published:** 2024-11-14

**Authors:** Yaser Shabanpour, Behnam Hajipour-Verdom, Parviz Abdolmaleki, Mozhgan Alipour

**Affiliations:** ^1^ Department of Biophysics, Faculty of Biological Sciences, Tarbiat Modares University, Tehran, Iran; ^2^ Functional Neurosurgery Research Center, Shohada Tajrish Comprehensive Neurosurgical Center of Excellence, Shahid Beheshti University of Medical Sciences, Tehran, Iran

**Keywords:** ferroptosis, oxidized membrane, realistic membrane, hydroperoxide derivatives, molecular dynamics simulation

## Abstract

Ferroptosis is a regulated form of cell death characterized by iron-dependent lipid peroxidation of polyunsaturated fatty acids (PUFAs). Despite its significance, the precise molecular mechanisms underlying ferroptosis remain elusive, particularly concerning their impact on membrane properties. This study aimed to investigate the biophysical changes in plasma membranes due to lipid peroxidation during ferroptosis and their impact on the uptake of doxorubicin (DOX), a potent anticancer agent linked to ferroptosis. Using all-atom molecular dynamics simulations, we compared native red blood cell membranes (protein-free domains) with a ferroptosis model, in which PUFAs were replaced with hydroperoxide derivatives. Our findings reveal that the ferroptotic membrane exhibits decreased thickness and increased lipid area while maintaining overall integrity. The hydroperoxide groups localized in the disordered tail regions, enhancing tail mobility and facilitating hydrogen bonding. Lipid lateral diffusion was significantly altered, both layers of the ferroptotic membrane exhibited slower diffusion rates compared to the native membrane. Furthermore, lipid oxidation affected diffusion activation energies. Importantly, we found that DOX could penetrate the oxidized ferroptosis membrane with a lower free-energy barrier (∆G_PB_) of approximately 38 kJ.mol^−1^. Consequently, DOX’s permeability was approximately seven orders of magnitude higher than that of the native membrane. In summary, lipid peroxidation during ferroptosis induces extensive structural and dynamic changes, influencing membrane behavior and potentially offering insights that could inform future therapeutic strategies.

## Introduction

Ferroptosis is a regulated form of programmed cell death that is mechanistically distinct from other pathways such as apoptosis and necroptosis (Dixon et al., 2012). It results from an imbalance between a cell’s oxidant and antioxidant mechanisms, leading to excessive accumulation of polyunsaturated fatty acid hydroperoxides (PUFA-OOHs) within the plasma membrane (Kuang et al., 2020; Do et al., 2023; Chen et al., 2021a; Barayeu et al., 2023).

Reactive oxygen and nitrogen species (ROS/RNS) have been implicated in lipid peroxidation through both non-enzymatic and enzymatic pathways (Panasenko et al., 1995; Weidinger and Kozlov, 2015; Su et al., 2019). Non-enzymatic pathways involve the elevation of ROS/RNS levels, often as a result of the iron-mediated Fenton reaction (Fenton, 1894; Haber and Weiss, 1934). Enzymatic pathways, on the other hand, are mediated by enzymes such as lipoxygenase (LOX) and cyclooxygenase (COX), especially 15-lipoxygenase (15LOX) which demonstrates significant pro-ferroptotic peroxidation activity towards double-PUFA-PEs (Phosphatidylethanolamines containing two PUFA tails in sn1 and sn2) (Seiler et al., 2008; Ding et al., 2003; Yang et al., 2016; Kagan et al., 2021; Samovich et al., 2024).

Cellular antioxidant defenses include mevalonate-dependent mechanisms (Sun et al., 2023), as well as compounds like vitamin E (α-tocopherol) (Hu et al., 2021). Enzymes such as superoxide dismutase (SOD), catalase (CAT) (Chen et al., 2021b), ferroptosis suppressor protein 1 (FSP1) (Doll et al., 2019), calcium-independent phospholipase A2β (iPLA2B) (Sun et al., 2021), and inducible nitric oxide synthase (iNOS) (He et al., 2022) play crucial roles in modifying oxidative stress and protecting cells from lipid peroxidation damage. Of particular importance is glutathione peroxidase 4 (GPX4), which catalyzes the reduction of PUFA-OOHs to non-toxic alcohols (PUFAs-OH), effectively countering ferroptosis (Ursini et al., 1982; Liu et al., 2018; Yang et al., 2014; Moon et al., 2019).

**FIGURE 1:**
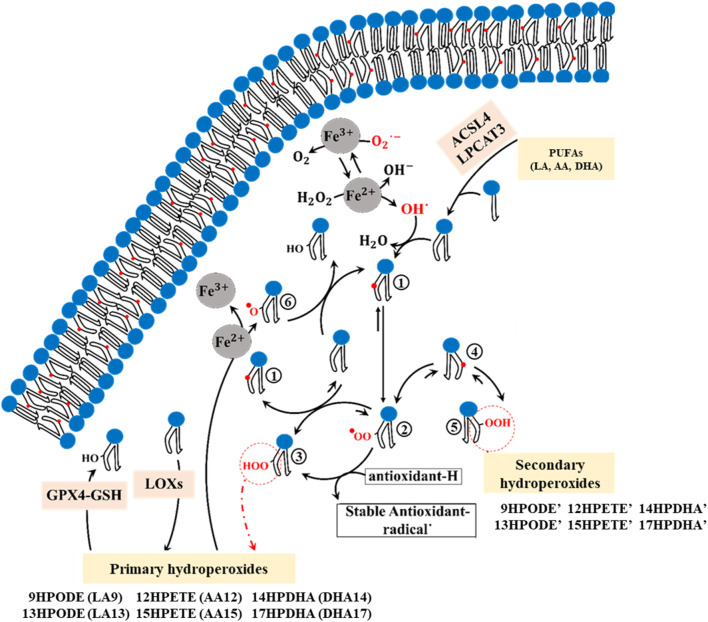
The chemical mechanism of PUFA peroxidation encompasses multiple sequential steps. 1; Formation of the cisoid pentadienyl radical, 2; Formation of the peroxyl radical, 3; Formation of the primary trans-cis hydroperoxides (9HPODE: 9-hydroperoxy-10E,11Z-octadecadienoic acid, 13HPODE: 13-hydroperoxy-9Z,11E-octadecadienoic acid, 12HPETE: 12-hydroperoxy-5Z,8Z,10E,14Z-eicosatetraenoic acid, 15HPETE: 15-hydroperoxy-5Z,8Z,11Z,13E-eicosatetraenoic acid, 14HPDHA: 14-hydroperoxy-4Z,7Z,10Z,12E,16Z,19Z-docosahexaenoic acid, 17HPDHA: 17-hydroperoxy-4Z,7Z,10Z,13Z,15E, 19Z-docosahexaenoic acid), 4; Formation of the transoid pentadienyl radical, 5; Formation of the Secondary trans-trans hydroperoxides (9HPODE’: 9-hydroperoxy-10E,12E-octadecadienoic acid, 13HPODE’: 13-hydroperoxy-9E,11E-octadecadienoic acid, 12HPETE’: 12-hydroperoxy-5Z,8E,10E,14Z-eicosatetraenoic acid, 15HPETE’: 15-hydroperoxy-5Z,8Z,11E,13E-eicosatetraenoic acid, 14HPDHA’: 14-hydroperoxy- 4Z,7Z,10E,12E,16Z,19Z-docosahexaenoic acid, 17HPDHA’: 17-hydroperoxy- 4Z,7Z,10Z,13E,15E, 19Z-docosahexaenoic acid), 6; Formation of the lipid alkoxyl.

Lipid peroxidation can alter the composition of the membrane and affect its physical properties, potentially causing damage (Neto and Cordeiro, 2016; Wong-Ekkabut et al., 2007). To understand how membrane composition is modified during ferroptosis, it is essential to investigate the mechanism of PUFA peroxidation (Figure 1). PUFAs are fatty acids containing two or more double bonds and at least one bis-allylic carbon atom (Wong-Ekkabut et al., 2007), serving as substrates for oxidation by ROS/RNS as well as LOXs. This oxidation involves hydrogen abstraction from bis-allylic carbon atoms, yielding a pentadienyl radical (PUFA°), which quickly interacts with molecular oxygen to generate a peroxyl radical (PUFA-OO°). These peroxyl radicals take hydrogen from other PUFAs, resulting in the formation of lipid hydroperoxides (PUFA-OOH) and the generation of new pentadienyl radicals. Lipid hydroperoxides then interact with Fe^2+^ to produce lipid alkoxyl radicals, which in turn abstracts hydrogen from adjacent PUFAs, thus propagating the radical chain reaction. Alternatively, they may take hydrogen from antioxidants, convert them into stable oxidized forms, and produce new hydroperoxides-a process known as the termination pathway (Porter, 1986; Marnett, 1987). In the presence of electron donors such as antioxidants (e.g., alpha-tocopherol) and other PUFAs, peroxyl radicals predominantly yield primary hydroperoxides with a trans(E)-cis(Z) configuration. However, in their absence, the primary hydroperoxide undergoes β-fragmentation to form secondary hydroperoxides with a trans(E)-trans(E) configuration (Schneider, 2009) (Figure 1).

The enzymes acyl-CoA synthetase long-chain family member 4 (ACSL4) and lysophosphatidylcholine acyltransferase 3 (LPCAT3) are required for inducing ferroptosis in cells (Kagan et al., 2017; Li et al., 2020). ACSL4 prepares PUFAs, particularly arachidonic acid (AA, 20:4 n- 5Z,8Z,11Z, 14Z) and linoleic acid (LA, 18:2 n-9Z, 12Z), for incorporation into membrane phospholipids by the LPCAT3 enzyme (Yuan et al., 2016; Doll et al., 2017; Wang and Tontonoz, 2019; Küch et al., 2014). These PUFAs are classified into omega-3 and omega-6 groups, both of which play crucial roles in inducing ferroptotic cell death (Trostchansky and Rubbo, 2019). While both LA (omega-6) and AA (omega-6) can be oxidized by ROS, AA serves as the primary substrate of LOX enzymes (Kagan et al., 2017; Trostchansky and Rubbo, 2019; Vickers, 2017; Ito et al., 2019; Shah et al., 2018). Notably, in human red blood cell plasma membranes, the three most common PUFAs are LA, AA, and docosahexaenoic acid (DHA, 22:6 n-4Z,7Z,10Z,13Z,16Z, 19Z) (Yawata, 2006). During ferroptosis, these PUFAs become susceptible to autoxidation by iron, as well as oxidation through LOX and COX enzymes, and ROS/RNS (Trostchansky and Rubbo, 2019). A variety of hydroperoxide isomers can be produced through both autoxidation and enzymatic oxidation of LA, AA, and DHA, including LA-9HPODE (hydroperoxyoctadecadienoic acid: 9OOH, 10E, 12Z), LA-13HPODE (hydroperoxyoctadecadienoic acid: 13OOH, 9Z, 11E), AA-12HPETE (hydroperoxyeicosatetraenoic acid: 12OOH, 5Z, 8Z, 10E, 14Z), AA-15HPETE (hydroperoxyeicosatetraenoic acid: 15OOH, 5Z, 8Z, 11Z, 13E), DHA-14HPDHA (Hydroperoxydocosahexaenoic acid: 14OOH, 4Z, 7Z, 10Z, 13Z, 15E, 19Z), and DHA-17HPDHA (Hydroperoxydocosahexaenoic acid: 17OOH, 4Z, 7Z, 10Z, 12E, 16Z, 19Z) (Trostchansky and Rubbo, 2019).

The lipid composition and the asymmetric distribution of lipids between membrane layers significantly influence the physical properties and functions of a membrane (Moon et al., 2019; van Meer, 2011). This distribution is regulated by proteins known as flippases and floppases in human red cell membranes (Marquardt et al., 2015; Devaux et al., 2008; Bevers et al., 1999), leading to an enrichment of phosphatidylcholine (PC) and sphingomyelin (SM) in the outer layer, and phosphatidylethanolamine (PE) and phosphatidylserine (PS) in the inner monolayer (Verkleij et al., 1973; Mohandas and Gallagher, 2008). While the cholesterol content of mammalian cell membranes is known to be approximately 50% of the phospholipid content, the exact distribution of cholesterol between bilayers remains debated (Yawata, 2006; Marquardt et al., 2015; Marquardt et al., 2016; Choubey et al., 2013).

This study aimed to investigate the intricate interplay between ferroptosis-induced lipid peroxidation and the dynamics of protein-free membranes. However, biological membranes typically exhibit a protein-to-lipid ratio of 1:50 to 1:100 (Ryan et al., 1988; Jeon et al., 2016), and protein crowding can significantly influence membrane structure and fluidity.The size of a protein’s hydrophobic region affects local lipid packing trough hydrophobic mismatch (Jensen and Mouritsen, 2004; Mitra et al., 2004); larger hydrophobic regions stretch surrounding lipids, decreasing their area per lipid (APL), while smaller regions have the opposite effect (Jensen and Mouritsen, 2004; Mitra et al., 2004). As protein concentration increases, APL generally decreases, and lipid lateral diffusion is reduced (Frick et al., 2007; Goose and Sansom, 2013), as normal Brownian motion shifts to non-Gaussian, anomalous diffusion patterns (Jeon et al., 2016; Javanainen et al., 2013). This crowding also leads to a reduction in membrane permeability (Jan Akhunzada et al., 2019).

In protein-free membranes, diffusion typically occurs on the scale of hundreds of nanoseconds (Javanainen et al., 2013) but in protein-crowded environments, diffusion slows dramatically, with normal diffusion beginning in the range of tens of microseconds and potentially extending to milliseconds in highly concentrated systems (Javanainen et al., 2013). This timescale exceeds the computational capacity of even the most advanced simulation systems.

Using molecular dynamics (MD) simulations, we examined the structural and dynamic alterations occurring within lipid membranes under ferroptotic conditions and assessed their impact on permeability to doxorubicin (DOX), a widely used chemotherapeutic agent. The connection between DOX and ferroptosis is in their association with ROS generation and PUFA peroxidation (Zhao et al., 2023; Hajipour Verdom et al., 2018; Micallef et al., 2020). Our study includes a range of modifications, including changes in membrane thickness, lipid mobility, and permeability, to elucidate the potential mechanisms underlying cellular responses to ferroptosis. Our findings provide valuable insights into the molecular basis of ferroptosis and its implications for cellular function and integrity. Understanding these processes is crucial for developing targeted interventions against ferroptosis-associated pathologies, making our study significant in the context of biotechnological and biomedical investigation.

## Models and methods

### Models Construction

The red blood cell plasma membrane was used to model both native and ferroptosis-induced membranes. This membrane’s lipid composition has been well-characterized by experimental techniques (Yawata, 2006; Yawata et al., 1984; Cooper, 1970; Van Deenen and De Gier, 1974). For the native membrane model, we used experimentally determined mole fractions of key phospholipid groups like PC, PE, PS, and SM. Fatty acid types and components for each group and membrane layer were selected based on Supplementary Data (Yawata, 2006) (Supplementary Table S1). To simplify the membrane model, phosphatidylinositol (Pi) was replaced with PS. Both Pi and PS contain a negative electric charge, though Pi constitutes a minor mole fraction in plasma membranes. Cholesterol, crucial for maintaining plasma membrane stability and function, was included at a molar ratio of 0.5 relative to total phospholipids, approximately reflecting its concentration in erythrocyte membranes (Yawata, 2006). Despite some studies suggesting symmetric cholesterol distribution between membrane layers and others indicating higher concentrations in the inner monolayer. We modeled symmetrical cholesterol distribution between the inner and outer layers to account for these uncertainties. The Phospholipid composition of the inner and outer layers was defined based on the details provided in Supplementary Tables S1, S2.

To derive the ferroptosis membrane, we replaced PUFA-containing phospholipids (PUFA-pls) of the native membrane with their hydroperoxide derivatives (Hpds) as described in Supplementary Table S3. Hydroperoxyl (OOH) groups were added to PUFA chains of phospholipids containing LA, AA, or DHA following experimental patterns shown in Supplementary Figures S1–S3. Since we did not find experimental data supporting the exact ratios of these oxidized isomers, for phospholipids containing solely LA, AA, or DHA in the sn-2 position, half were replaced with the Hpds 9HPODE (∼4.3 mol%), 12HPETE (∼5.6 mol%), or 14HPDHA (∼4 mol%), and the other half with 13HPODE (∼4.6 mol%), 15HPETE (∼5.6 mol%), or 17HPDHA (∼4 mol%), respectively. Phospholipids containing LA, AA, or DHA in sn-1/sn-2 positions incorporated isomers containing 13HPODE/9HPODE (∼1.6 mol%), 15-HPETE/12HPETE (∼2.3 mol%), or 17-HPDHA/14HPDHA (∼1.6 mol%), respectively. As observed, double hydroperoxidized phospholipids are present in low ratios in our modeled ferroptosis membrane. Recently Dr. Samovich et al. showed that these double hydroperoxidized phospholipids play a crucial role in ferroptosis induction (Samovich et al., 2024).

Supplementary Figures S1–S3 show the hydroperoxidized phospholipids containing HPODEs, HPETEs, and HPDHAs. The connectivity shapes, atom types in the CHARMM36 force field (Klauda et al., 2010; Bjelkmar et al., 2010; Piggot et al., 2012), and charge definitions of phospholipids with Hpds from both isomers of LAs, AAs, and DHAs are defined in Supplementary Tables S4–S6. The CHARM-GUI interface (Wu et al., 2014; Jo et al., 2008; Jo et al., 2009) was used to model the native membrane composition based on Supplementary Table S2. The ferroptosis membrane was constructed using the Packmol package (Martínez et al., 2009), maintaining a similar composition to the native membrane but substituting phospholipids containing LA, AA, and DHA with their Hpds. The distance at which the molecules’ atoms were allowed to assemble around each other in Packmol was set by the tolerance parameter, which was 2 Å. Simulation boxes containing 200 phospholipids and 100 cholesterol molecules were generated for both membranes using parameters listed in Supplementary Table S7.

#### Molecular dynamics simulations

We performed all-atom MD simulations using GROMACS (version 2018.1) (Abraham et al., 2018) and the CHARMM36 force field to simulate four MD simulations: two simulations for the native membrane (n = 2) and two for the ferroptosis membrane (n = 2). The reported results of each modeled membrane are the average of the two related simulations. Graphs were plotted using the xmgrace plotting tool (Cowan and Grosdidier, 2000) and MD snapshots of the membranes were analyzed using VMD (Visual Molecular Dynamics) software (Humphrey et al., 1996).

Initially, energy minimization for all systems was conducted using the steepest descent minimization algorithm over 100,000 steps. The energetically minimized systems were then equilibrated in the NVT ensemble using the leap-frog integrator in a two-step equilibration process (Bussi et al., 2007; Alipour et al., 2023). The first equilibration step was performed for 1500 picoseconds (ps) at 305 K (K), followed by a second equilibration step for 2500 ps at 310 K. During NVT equilibration, a time step of 2 femtoseconds (fs) was used, and temperature coupling was achieved with the velocity rescale thermostat (Bussi et al., 2007).

Subsequent NPT ensemble equilibration involved semi-isotropic pressure control at 1 bar using the Parrinello-Rahman barostat (Parrinello and Rahman, 1981) with a time step of 2 fs. The pressure was adjusted in four steps: 1250, 1500, 2500, and 5000 ps, while maintaining a constant temperature of 310 K. All restraints were removed in the final step of the NPT equilibration. Throughout the equilibration and production steps, the Verlet list update method was used as the cutoff scheme, with the neighbor list updated every 20 steps. The cutoff distance for the neighbor list (rlist) was set to 1.2 nm (nm). For Lennard-Jones and short-range electrostatic interactions, a cutoff of 1.2 nm was applied, and long-range electrostatic interactions were computed using the particle mesh Ewald (PME) method (Cheatham et al., 1995).

The production step of the simulations was performed for 500 ns (n = 2) using a time step of 2 fs, a temperature of 310 K, and a pressure of 1 bar. The leap-frog algorithm was employed to integrate Newton’s equations of motion during this step. The root-mean-square deviation (RMSD) of the systems reached a steady state, indicating that the simulated structures were relatively stable. Additionally, it was observed that the n1 and n2 simulations of each native and ferroptosis membrane showed a high degree of similarity (Supplementary Figure S4).

#### Deuterium order parameter (S_CD_)

We calculated the deuterium order parameter S_CD_ (Piggot et al., 2017) to obtain the rotational diffusion (motional order) of the acyl chains relative to the membrane normal (*z*-axis). A low S_CD_ value indicates that the acyl chains have high motional freedom, while a high S_CD_ value suggests a rigid, crystal-like membrane state with limited freedom of motion (Yeagle, 2016). An S_CD_ = 1 indicates that lipid tails are aligned along the membrane normal, whereas an S_CD_ = −0.5 indicates perpendicular orientation (Yeagle, 2016). The S_CD_ can be experimentally obtained through techniques like electron spin resonance (ESR) and ^2^H-^13^C NMR, as well as through simulations (Yeagle, 2016; Vermeer et al., 2007). In MD simulations, S_CD_ calculates the orientation of C-H bonds relative to the membrane normal (Yeagle, 2016; Karami and Jalili, 2015), using the following Equation 1.

$$S_{\text{CD}}=\frac{1}{2}\langle 3\;\cos^{2}\left(\theta_{j}\right)‐1\rangle$$ (1)

Where θj is the angle between the Cj−1 and Cj+1 atoms axes and the membrane normal, which defines the orientation of the molecule within the bilayer.

#### Lipids lateral diffusion

The lateral diffusion coefficient (D) of lipids reflects the Brownian motion associated with the exchange of lipids within the membrane (Wong-Ekkabut et al., 2007). To obtain D values, we first calculated the mean-square displacements (MSD) using Equation 2 and then applied the Einstein relation (Equation 3). Here, n_species_ represents the number of lipid species, and r_i_ (t) denotes the position of lipid i at time t. Before MSD calculation, we subtracted the motion of the monolayers and the center of mass of the membranes.

$$MSD=\frac{1}{n_{Species}}\sum_{i=1}^{n_{species}}\langle {\left[r_{i}\left(t\right)-r_{i}\left(t=0\right)\right]}^{2}\rangle$$ (2)

$$D_{lateral}=\frac{1}{4}\;\lim_{t\to \infty }\frac{d}{dt}\;\left(MSD\right)$$ (3)

Two important factors influencing D are the free area theory and the activation energy barrier of Brownian motion (Falck et al., 2004). The free area theory relates lateral mobility to the available free area per molecule ( $a_{f}$ ) and the lipid packing, represented by the cross-sectional area per lipid ( $a_{l}$ ) as shown in Equation 4. The activation energy barrier, described by the Boltzmann relation, must be overcome for diffusion to occur between sites.

$$D\;∼\;e^{-\frac{a_{l}}{a_{f}}}$$ (4)

#### Force field parameters

The topological parameters for phospholipids, cholesterol, water, and ions were obtained from the CHARMM36 force field. The TIP3P water molecules were utilized for the simulation. To identify the OOH groups of oxidized phospholipids, additional parameters were used from CGenFF (Vanommeslaeghe et al., 2010) and Julian Garrec’s work (Garrec et al., 2014). These parameters were calculated based on the B3LYP/6-31G* quantum mechanics theory using Gaussian G9 and the FFTK program. The hydroperoximethane (CH3COOH) was employed as the model molecule. Parameters for bond stretching, angle, dihedral angles, and charge parameters were determined accordingly (Supplementary Tables S8–10).

#### Potential of mean force (PMF)

We used the umbrella sampling method (Torrie and Valleau, 1977) to calculate the pure free-energy barrier (∆GPB) or the potential of mean force (PMF) for the passage of DOX across the four systems comprising two modeled membranes. The topology parameters of DOX were provided by CGenFF. Initially, we obtained the initial structures from steered molecular dynamics (SMD) (Isralewitz et al., 2001), where DOX was moved with a force constant of 100 kJ.mol^−1^.nm^−2^ and a velocity of 0.001 nm.ps^−1^ from the outer aqueous phase (z = 4.0 nm) to the inner aqueous phase (z = −4.0 nm) through the membrane models selected from 500 ns simulation (n = 2). We divided this process into 40 windows, each 0.2 nm apart within our membranes. Each window was simulated for 20 ns in the NPT ensemble, with the first 10 ns serving as the equilibrium phase. Subsequently, we generated the PMF profiles using the WHAM analysis method (Kumar et al., 1992).

Furthermore, we utilized the inhomogeneous solubility-diffusion model (Marrink and Berendsen, 1994; Dickson et al., 2017) to calculate the permeability coefficient of DOX in membrane models. The permeability coefficient was determined using Equation 5, which considers the ∆G_PB_ and the diffusion coefficient (D) of DOX at specific z-positions within the model membranes. The diffusion coefficient can be calculated using the force autocorrelation method (Marrink and Berendsen, 1994). Here, R represents the ideal gas constant (8.314 J.mol^−1^.K^−1^) and T denotes the temperature (298 K).

$$P={\left[\int_{{-4}_{nm}}^{4_{nm}}\frac{e^{{∆G}_{PB\left(z\right)}\frac{1}{RT}}}{D\left(z\right)}\right]}^{-1}$$ (5)

## Results and discussioni

### Mass density profiles

**FIGURE 2:**
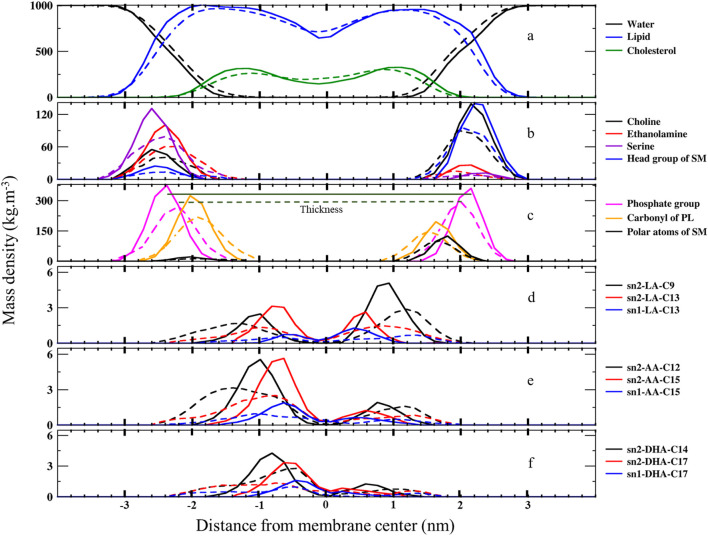
The mass density profiles along the bilayer normal (*z*-direction) from the center of the membranes over the last 400 ns (n = 2) for **(A)** water, lipids, and cholesterol, **(B)** lipid head groups, **(C)** carbonyl and phosphate groups, and **(D)** carbons bonded to OOH moieties in LAs, AAs, and DHAs. The solid lines represent the native membrane, while the dashed lines represent the ferroptosis membrane.

**FIGURE 3:**
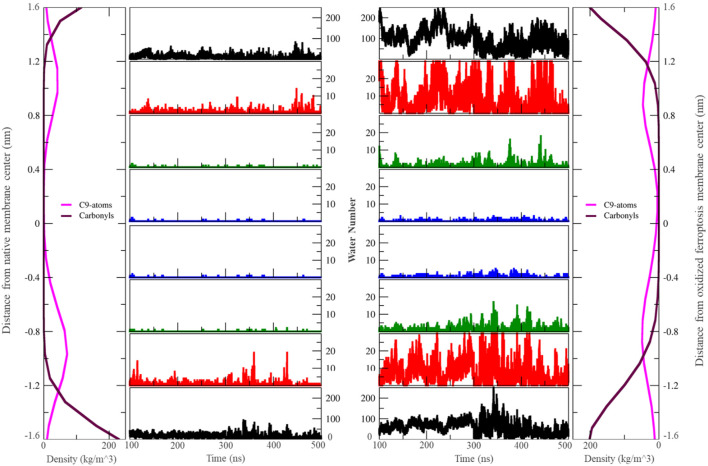
The number of water molecules located in different zones around the center of the membrane varies over the last 400 ns (n = 2). Each zone is separated by 0.4 nm. In this context, “carbonyls” refer to the carbonyl groups of phospholipids, and ‘C9-atoms’ are associated with the ninth carbon atom in the lipid tails (left: native membrane, right: oxidized ferroptotic membrane).

We calculated the mass density profile of individual components as a function of distance from the membrane center for both native and ferroptosis membranes, illustrating their positions and water permeation (Figures 2, 3; Supplementary Tables S1, S12). Figures 2A, 3 demonstrate that water molecules penetrated deeper into the ferroptosis membrane, indicating increased water permeability. However, despite heightened permeability, the water density at the center of the membrane is negligible, suggesting that the membrane’s integrity was maintained under ferroptosis conditions. This finding aligns with other studies that did not observe pore formation when bilayers underwent hydroperoxidation (Jurkiewicz et al., 2012; Weber et al., 2014).

The minimal lipid density at the center (Z ∼ 0) denotes the random packing of lipid chains. We also found slightly increased lipid density in the central region of the ferroptosis membrane, potentially due to the interdigitated lipids from different layers (Wong-Ekkabut et al., 2007; Kumar et al., 2018; Mason et al., 1997). Furthermore, we observed another structural modification: in the native membrane, cholesterol resided near the tails proximate to the polar interface, while in the ferroptosis membrane, cholesterol shifted toward the center.

Moreover, the density profiles of choline, serine, ethanolamine, and head group of SM in the inner layer of the ferroptosis membrane shifted approximately 0.2 nm, 0.4 nm, 0.4 nm, and 0.4 nm toward the center, respectively, compared to the native membrane. Similarly, in the outer layer, these profiles shifted approximately 0.2 nm, 0.4 nm, 0.2 nm, and 0.4 nm toward the center, respectively, with peak positions 0.2 nm closer to the core. These results indicated that the ferroptosis membrane is generally thinner than the native membrane (Figure 2B; Supplementary Table S12).

In addition, the density profiles of polar atoms such as phosphates, carbonyls, and polar atoms of SMs (-CO-NH-C-C-OH), shifted 0.2 nm toward the core in both layers of the ferroptosis membrane compared to the native membrane. Their peak positions were closer to the core by approximately 0.17 nm, 0.1 nm, and 0.1 nm in the inner layer and by about 0.15 nm, 0.1 nm, and 0.2 nm in the outer layer (Figure 2C; Supplementary Tables S11, S12).

Furthermore, Figure 2D shows that the density profiles for carbon atoms with OOH groups in the ferroptosis membrane were more widely distributed toward the polar interface compared to the native membrane. In LAs, oxidized C13 in both sn1 and sn2 positions were more widely distributed than oxidized C9 in sn2. Similarly, in AAs, oxidized C15 in sn1 and sn2 were more widely distributed than oxidized C12 in sn2. In DHAs, oxidized C17 in sn1 and sn2 were more widely distributed than oxidized C14 in sn2. These findings suggest that OOH groups closer to the chain ends induce more significant structural modifications by moving more strongly toward the polar interface. This phenomenon aligns with the Whisker model proposed for the structure of oxidized cell membranes (Sun et al., 2021; Greenberg et al., 2008).

Supplementary Table S13 presents the thickness and area per lipid of our simulations, alongside other experimental and simulation results for comparison. We determined membrane thickness by measuring the distance between phosphate peaks in the inner and outer layers (Supplementary Table S11; Figure 2C). Recent studies using high-resolution X-rays to obtain structural properties of erythrocyte membrane domains identified two states: ordered (lipid tails in all-trans configurations) and disordered (bent lipid chains) (Himbert et al., 2017). The ordered domain thickness was 4.6 nm, which is close to our 4.52 nm native membrane simulation result. Meanwhile, the disordered domain thickness was 4.1 nm, similar to our 4.2 nm ferroptosis membrane simulation. These measurements indicate a 0.32 nm decrease in thickness from the native to the ferroptosis membrane. The thicknesses of the inner and outer layers of the native and ferroptosis membranes were 2.37/2.15 nm and 2.2/2.0 nm, respectively. Notably, the outer layer was thinner than the inner layer in both membranes. Results indicate that ferroptosis decreased the thickness of inner/outer layers by 0.17/0.15 nm. Additionally, the inner and outer layers of the ferroptosis membrane had a 0.06 nm^2^ larger area per lipid compared to the native membrane. These results are consistent with previous studies (Wong-Ekkabut et al., 2007; Himbert et al., 2017; Yang et al., 2020) shown in Supplementary Table S13 (see Supplementary Material for more discussion), which confirm the reliability of our all-atom force field parameters in defining HPds in the membrane. Our model membranes also closely match to experimental thickness and area results.

The increased lipid surface area in the ferroptosis membrane is attributed to interactions between the oxide groups and the polar interface, as well as the bending of lipid chains. Other studies also observe hydroperoxide groups near the polar interface (Garrec et al., 2014). This increase in lipid surface area is a notable structural change that can significantly impact membrane dynamics and function.

### Lipid-order parameter

**FIGURE 4:**
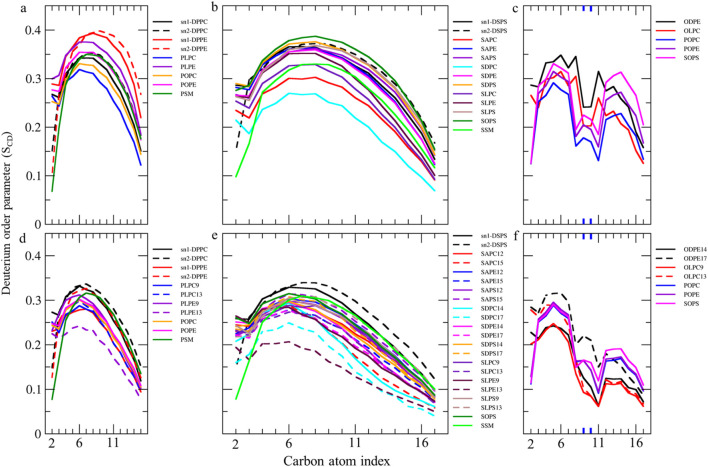
Deuterium order parameter S_CD_ profiles over the last 400 ns (n = 2) for PALs, SAs, and OAs in the **(A–C)** native and **(D–F)** ferroptosis membranes. The carbon index ranges from the carbonyl carbon (+1) to the penultimate carbon. The carbons participating in double bonds are labeled in blue and shown as thicker lines.

**FIGURE 5:**
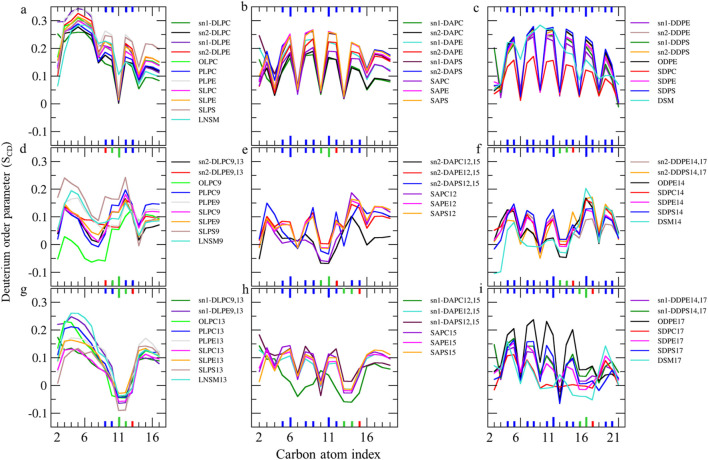
**(A–C)** Deuterium order parameter S_CD_ profiles over the last 400 ns (n = 2) for LAs, AAs, and DHAs in the native membrane. **(D–I)** The S_CD_ profiles of their hydroperoxide products, such as LAs9, AAs12, DHAs14, LAs13, AAs15, and DHAs17 for the ferroptosis membrane. The carbon index ranges from the carbonyl carbon (+1) to the penultimate carbon. The carbons participating in cis and trans double bonds are labeled in blue and green, respectively, and shown as thicker lines. Carbons carrying the OOH moiety are marked in red and shown as thicker lines.

We measured S_CD_ values for all carbon atoms in individual fatty acids to compare their motional freedom in detail (Figures 4, 5). The order parameter profile exhibits a plateau state for palmitic acids (PAs, 16:0, S_CD_ ∼0.3–0.4) and stearic acids (SAs, 18:0, S_CD_ ∼0.23–0.38) at different sn1 and sn2 positions in various lipids, from C4 to C9 (Figures 4A,B). The S_CD_ then rapidly decreases down the chain to its lowest value, aligning with results from spectroscopic techniques (Vermeer et al., 2007; Galiullina et al., 2019). This indicates that the hydrocarbon chain near the glycerol backbone has low motional freedom and is more aligned with bilayer normal, while the core region exhibits greater fluidity with low alignment with bilayer normal. The S_CD_ order parameter for all carbons in PAs and SAs shifts to lower values in the ferroptosis membrane (Figures 4D,E), suggesting that even non-oxidized tails in ferroptosis membranes have greater fluidity compared to the native membrane. Meanwhile, Figure 4C shows the order parameter profile for oleic acids (OA, C18:1 n-9) in the native membrane, exhibiting a plateau state (S_CD_ ∼0.25–0.35) from C3-C7, rapidly dropping to its lower value around double bond, C9-C10, before rising once more from C12-C15, as experimentally confirmed (Karami and Jalili, 2015; Hoopes et al., 2011). This demonstrates that the presence of a double bond on C9-C10 in OAs causes a decrease in fluidity in the end portion below the double bond, compared to stearic acids.

Furthermore, the order parameter for the carbon atoms in OAs shifts to lower values in the ferroptosis membrane (Figure 4F), suggesting that monounsaturated tails have greater fluidity compared to the native membrane. This increased fluidity in both saturated and monounsaturated fatty acid chains in the ferroptosis membrane indicates a significant alteration in the membrane’s physical properties under ferroptotic conditions. This could have substantial implications for membrane permeability and the overall cellular response during ferroptosis.

The order parameter of PUFAs is illustrated in Figure 5. In Figure 5A, the profile for LAs in the native membrane shows a plateau state from C3 to C7 (S_CD_ ∼0.22–0.35). The S_CD_ then drops at C8, increases at the double bond (C9-C10), drops again at C11, increases at the second double bond (C12-C13), decreases at C14, and finally increases again towards the chain end. This pattern indicates that the double bonds in LAs reduce fluidity in the tail end compared to stearic acids, resembling the effect of the double bond in OAs.

Additionally, Figures 5D,G shows the order parameter for hydroperoxidized LAs isomers, specifically 9HPODE (LA9) and 13HPODE (LA13), respectively. For LA9, the order parameter reduces from C3 to C9 compared to the native membrane due to the oxidation at C9. However, the order parameter increases at C11 due to the formation of a new trans double bond. For LA13, the order parameter significantly reduces from C9 to C13 due to the oxidation at C13. Consequently, hydroperoxidation at C9 disrupts the order in the region proximal to the glycerol backbone, while oxidation at C13 disrupts the order in the mid-chain region.

As shown in Figures 5B,C, the order parameter profiles in the native membrane for AA and DHA in phospholipids exhibit frequent rises and drops, with high order parameters at double bond carbons and low order parameters between continuous double bonds consistent with previous findings (Hyvönen and Kovanen, 2005; Saiz and Klein, 2001). Figures 5E,H, shows significantly reduced order parameters for hydroperoxidized AA isomers 12HPETE (AA12) and 15HPETE (AA15) compared to native membrane. The most disordered regions localize to the hydroperoxide moiety, specifically spanning C10 to C12 for AA12 and C13 to C15 for AA15. Similarly, Figures 5F,I, show significantly reduced and disturbed order parameters for hydroperoxidized DHA isomers 14HPDHA (DHA14) and 17HPDHA (DHA17) compared to the native membrane. The most disordered regions for DHA14 span C13 to C14 and for DHA17 span C16 to C17, indicating the localization of disorder around the hydroperoxide groups.

**FIGURE 6:**
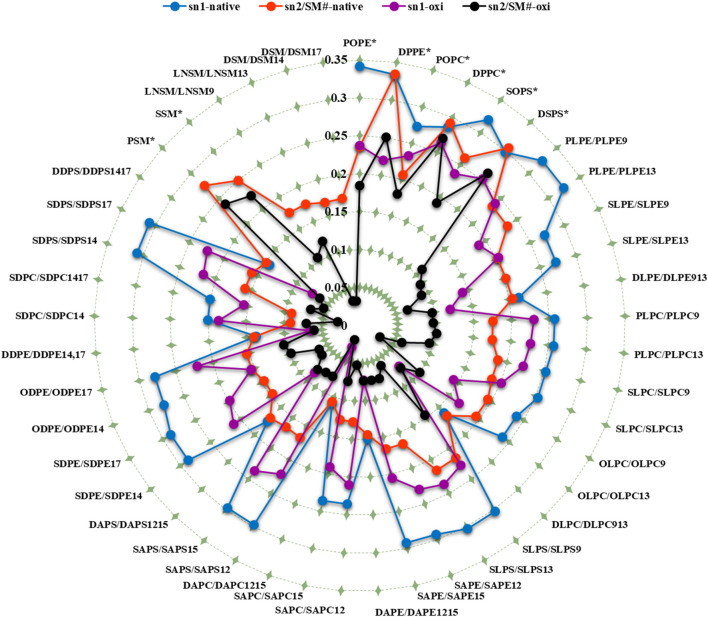
The average deuterium order parameter (denoted as <S_CD_>) over the last 400 ns (n = 2) for the sn-1 and sn-2 chains of phospholipids, as well as the fatty acid tails of the sphingomyelins (SM#), is compared between the native and ferroptosis membranes over the last 400 ns. For instance, the notation “PLPE/PLPE9” indicates that PLPE is present in the native membrane, while PLPE9, its oxidized isoform, is found in the ferroptosis membrane. The symbol “^*^” denotes non-oxidized lipids that exist in the ferroptosis membrane like their presence in the native membrane. The term “oxi” in the figure legend refers to the S_CD_ of lipid tails in the ferroptosis membrane.

In addition to the individual S_CD_ values, the average S_CD_ of each fatty acid in sn1 and sn2 of all phospholipids was plotted for both membranes (Table 1; Figure 6). The average S_CD_ of nearly all oxidized and non-oxidized lipid tails in sn1 and sn2 decreased in the ferroptosis membrane compared to the native membrane, suggesting that the ferroptosis membrane is generally more fluid than the native membrane.

**TABLE 1:** The average deuterium order parameter (S_CD_) and standard deviation (SD) values over the last 400 ns (n = 2) for the sn-1 and sn-2 chains of phospholipids, as well as the fatty acid tails of the sphingomyelins (SM^#^) in the native and ferroptosis membranes.

Phospholipids	S_CD_			
	Native membrane		Ferroptosis membrane	
	sn1	sn2/SM^#^	sn1	sn2/SM^#^
	Avg±SD	Avg±SD	Avg±SD	Avg±SD
PLPE	0.32 ± 0.05	0.23 ± 0.08	—	—
PLPE9	—	—	0.24 ± 0.06	0.11 ± 0.04
PLPE13	—	—	0.19 ± 0.05	0.12 ± 0.07
SLPE	0.27 ± 0.07	0.20 ± 0.07	—	—
SLPE9	—	—	0.20 ± 0.06	0.09 ± 0.05
SLPE13	—	—	0.14 ± 0.05	0.06 ± 0.04
DLPE	0.21 ± 0.08	0.20 ± 0.08	—	—
DLPE9,13			0.12 ± 0.09	0.10 ± 0.03
PLPC	0.26 ± 0.05	0.18 ± 0.07	—	—
PLPC9	—	—	0.23 ± 0.05	0.10 ± 0.06
PLPC13	—	—	0.23 ± 0.06	0.10 ± 0.08
SLPC	0.25 ± 0.07	0.19 ± 0.07	—	—
SLPC9	—	—	0.22 ± 0.06	0.09 ± 0.04
SLPC13	—	—	0.20 ± 0.06	0.06 ± 0.07
OLPC	0.24 ± 0.05	0.19 ± 0.08	—	—
OLPC9	—	—	0.14 ± 0.0	0.03 ± 0.03
OLPC13	—	—	0.16 ± 0.08	0.10 ± 0.07
DLPC	0.16 ± 0.07	0.16 ± 0.07	—	—
DLPC9,13			0.07 ± 0.05	0.07 ± 0.04
SAPE	0.29 ± 0.07	0.17 ± 0.08	—	—
**SAPE12**	—	—	0.23 ± 0.06	0.07 ± 0.05
SAPE15	—	—	0.20 ± 0.06	0.07 ± 0.04
DAPE	0.15 ± 0.06	0.14 ± 0.06	—	—
DAPE12,15			0.07 ± 0.04	0.07 ± 0.04
SAPC	0.24 ± 0.06	0.13 ± 0.05	—	—
**SAPC12**	—	—	0.21 ± 0.06	0.05 ± 0.07
SAPC15	—	—	0.19 ± 0.07	0.07 ± 0.07
DAPC	0.11 ± 0.05	0.11 ± 0.05	—	—
DAPC12,15			0.03 ± 0.06	0.02 ± 0.05
SLPS	0.30 ± 0.06	0.22 ± 0.07	—	—
SLPS9	—	—	0.23 ± 0.07	0.15 ± 0.06
SLPS13	—	—	0.24 ± 0.06	0.06 ± 0.08
SAPS	0.3 ± 0.06	0.17 ± 0.08	—	—
SAPS12	—	—	0.22 ± 0.07	0.08 ± 0.05
SAPS15	—	—	0.24 ± 0.06	0.08 ± 0.04
DAPS	0.18 ± 0.07	0.17 ± 0.08	—	—
DAPS12,15			0.09 ± 0.06	0.08 ± 0.06
SDPE	0.29 ± 0.07	0.15 ± 0.09		
SDPE14	—	—	0.21 ± 0.07	0.06 ± 0.05
SDPE17	—	—	0.20 ± 0.06	0.06 ± 0.05
ODPE	0.28 ± 0.05	0.15 ± 0.10	—	—
ODPE14	—	—	0.15 ± 0.06	0.09 ± 0.06
ODPE17	—	—	0.22 ± 0.07	0.10 ± 0.08
DDPE	0.14 ± 0.08	0.14 ± 0.08	—	—
DDPE14,17			0.07 ± 0.04	0.06 ± 0.04
SDPC	0.20 ± 0.06	0.09 ± 0.05	—	—
SDPC14	—	—	0.18 ± 0.07	0.07 ± 0.05
SDPC17	—	—	0.16 ± 0.07	0.03 ± 0.04
SDPS	0.31 ± 0.06	0.16 ± 0.10	—	—
SDPS14	—	—	0.22 ± 0.06	0.07 ± 0.05
SDPS17	—	—	0.22 ± 0.07	0.05 ± 0.07
DDPS	0.14 ± 0.09	0.15 ± 0.09	—	—
DDPS14,17	—	—	0.08 ± 0.05	0.06 ± 0.05
POPE	0.30 ± 0.05	0.24 ± 0.05	0.24 ± 0.05	0.18 ± 0.06
DPPE	0.34 ± 0.05	0.33 ± 0.07	0.22 ± 0.05	0.25 ± 0.06
POPC	0.27 ± 0.05	0.21 ± 0.05	0.23 ± 0.05	0.18 ± 0.06
DPPC	0.29 ± 0.05	0.29 ± 0.06	0.26 ± 0.05	0.26 ± 0.06
DSPS	0.30 ± 0.06	0.31 ± 0.06	0.25 ± 0.07	0.26 ± 0.07
SOPS	0.32 ± 0.06	0.26 ± 0.06	0.24 ± 0.07	0.19 ± 0.05
PSM^#^	—	0.28 ± 0.07	—	0.24 ± 0.07
SSM^#^	—	0.25 ± 0.07	—	0.22 ± 0.07
LNSM^#^	—	0.18 ± 0.07	—	—
LNSM9^#^	—	—	—	0.11 ± 0.04
LNSM13^#^	—	—	—	0.12 ± 0.09
LSM^#^	—	0.21 ± 0.09	—	0.15 ± 0.09
BSM^#^	—	0.22 ± 0.06	—	0.21 ± 0.09
DSM^#^	—	0.17 ± 0.07	—	—
DSM14^#^	—	—	—	0.03 ± 0.07
DSM17^#^	—	—	—	0.03 ± 0.05
NSM^#^	—	0.17 ± 0.11	—	0.16 ± 0.11

SM^#^ refers to the deuterium order parameter (S_CD_) for the fatty acid tails of sphingomyelins.

Additionally, the average S_CD_ values of the same fatty acids in both sn1 and sn2 positions were calculated (Table 2). Results show that the average S_CD_ of identical fatty acids in the sn1 and sn2 positions are nearly equal in both native and ferroptosis membranes, indicating motional degrees of freedom of acyl chains are independent of their position on phospholipids (see Supplementary Material for more discussion). Furthermore, although average S_CD_ values were identically high for PAs and SAs, they successively decreased from PAs and SAs to OA, LA, AA, and DHA. This decrease results from kinks in polyunsaturated chains creating more free space, preventing the dense adjacent tail packing, and enabling greater freedom of movement.

**TABLE 2:** The average S_CD_ for similar fatty acids in the sn-1, sn-2, and sphingomyelin over the last 400 ns (n = 2).

Membrane		S_CD_																	
		PA			STA			OLA			LA			AA			DHA		
		sn1	sn2	SM	sn1	sn2	SM	sn1	sn2	SM	sn1	sn2	SM	Sn1	sn2	SM	sn1	sn2	SM
Native	Avg SD	**0.30** **0.06**	**0.31** **0.07**	**0.28** **0.07**	**0.28** **0.07**	**0.30** **0.07**	**0.25** **0.07**	**0.25** **0.05**	**0.23** **0.05**	-	**0.19** **0.09**	**0.19** **0.08**	**0.18** **0.07**	**0.14** **0.07**	**0.14** **0.07**	**-**	**0.14** **0.08**	**0.14** **0.09**	**0.17** **0.07**
Ferroptosis	**Avg** **SD**	**0.23** **0.06**	**0.26** **0.06**	**0.24** **0.07**	**0.21** **0.07**	**0.26** **0.07**	**0.22** **0.07**	**0.17** **0.07**	**0.18** **0.06**	**-**	**0.09** **0.08**	**0.09** **0.06**	**0.11** **0.08**	**0.06** **0.05**	**0.06** **0.05**	**-**	**0.07** **0.05**	**0.07** **0.06**	**0.03** **0.06**

Figures 4, 5 demonstrate that when a polyunsaturated fatty acid occupies the sn2 position, the sn1 fatty acid exhibits a lower order compared to when a saturated fatty acid is in the sn2 position. This indicates that the presence of polyunsaturated chains in the sn2 position disrupts the ordering of the sn1 chains more than saturated chains do. Moreover, some phospholipids exhibit significant variations in the order parameters of the same acyl chains between native and ferroptosis membranes. This variation likely stems from the influence of nearby lipids, especially cholesterol, which plays a crucial role in modulating membrane order and dynamics (Yeagle, 2016) [see Supplementary Material for more discussion (Supplementary Figure S5)].

Overall, our results indicate that fatty acid oxidation increases the motional freedom of both oxidized and adjacent non-oxidized acyl chains. This heightened motional freedom in the membrane interior can enhance permeability to small molecules. High-ordered membranes are generally less permeable to water and other small molecules. Therefore, the increased fluidity and reduced order parameters observed in ferroptosis membranes suggest higher permeability compared to native membranes. Additionally, the variations in acyl chain order parameters arise from their proximity to cholesterols and other low-ordered chains. These findings highlight the critical roles of fatty acid oxidation, lipid composition, and cholesterol content in modulating membrane properties and permeability.

### Lipid tails geometry

**FIGURE 7:**
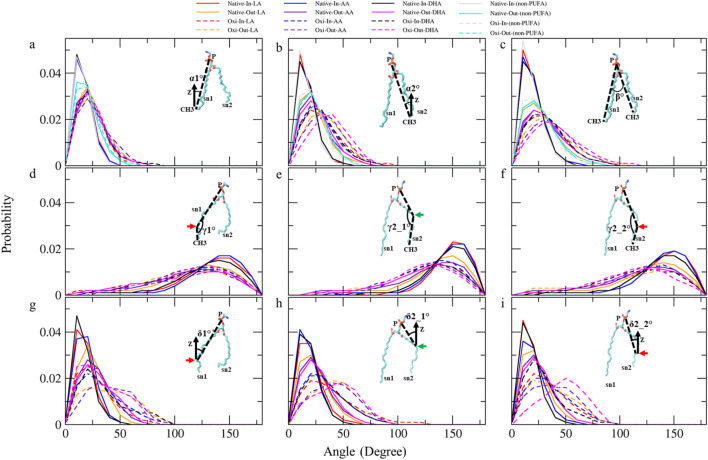
The analyses of angle distribution of lipid tails over the last 400 ns (n = 2) using various angles. Each angle is defined between two vectors (v1 and v2) representing different parts of the lipid tail. **(A)** α1° (v1: (P)-(sn1-CH3) and v2: (*Z*-axis)), **(B)** α2°(v1: (P)-(sn2-CH3) and v2: (*Z*-axis)), **(C)** β° (v1: (P)-(sn1-CH3) and v2: (P)-(sn2-CH3)), **(D)** γ1° (v1: (P)-(sn1-COOH) and v2: (sn1-COOH)-(sn1-CH3)), **(E)** γ2_1° and **(F)** γ2_2° (v1: (P)-(sn2-COOH) and v2: (sn2-COOH)-(sn2-CH3)), **(G)** δ1° (v1: (P)-(sn1-COOH) and v2: (*Z*-axis)), **(H)** δ2_1° and **(I)** δ2_2° (v1: (P)-(sn2-COOH) and v2: (*Z*-axis)).The angles capture the orientation and conformational changes of the lipid tails in the native and ferroptosis membranes. The term “Oxi” refers to the Ferroptosis membrane. (the green/red arrows refer to the carbons which carry the OOH group in oxidized tails at various positions (LA: C9, AA: C12, DHA: C14)/(LA: C13, AA: C15, DHA: C17)).

To determine the phospholipid structure, we analyzed the shapes of individual molecules by calculating the interior angles of lipid tails. We also calculated tilt angles representing tail and segment orientation relative to the membrane normal and membrane-water interface (Figure 7). Average angles were calculated for all phospholipid types in both native and ferroptosis membrane monolayers, including those with LAs (Supplementary Table S14), AAs (Supplementary Table S15), DHAs (Supplementary Table S16), and non-PUFAs (Supplementary Table S17). Furthermore, Table 3 shows average angles for four phospholipid groups (LA, AA, DHA, non-PUFA) in both monolayers using Supplementary Table Data. To analyze angle distributions, these four groups were examined in both native and ferroptosis membrane monolayers (Figure 7). This provided insights into phospholipid structural properties and membrane organization, detailed in the Supplementary Material.

**TABLE 3:** The average tilt and inner angles of phospholipids containing LAs, AAs, DHAs, and non-PUFA tails, and the average tilt angle of the cholesterol over the last 400 ns (n = 2). Each angle is defined between two vectors, v1 and v2.

Lipid tails /cholesterol	Membrane	Inner layer										Outer layer									
		α1°	α2°	α3°	β°	γ1°	γ2_1°	γ2_2°	δ1°	δ2_1°	δ2_2°	α1°	α2°	α3°	β°	γ1°	γ2_1°	γ2_2°	δ1°	δ2_1°	δ2_2°
LLA	Native	**19**	**13**	**-**	**21**	**139**	**150**	**142**	**25**	**16**	**14**	**21**	**20**	**-**	**27**	**116**	**136**	**126**	**28**	**20**	**19**
	Ferroptosis	**21**	**31**	**-**	**34**	**130**	**118**	**115**	**28**	**30**	**42**	**27**	**33**	**-**	**41**	**103**	**108**	**115**	**52**	**54**	**44**
AA	Native	**17**	**17**	**-**	**22**	**137**	**145**	**139**	**20**	**17**	**19**	**21**	**34**	**-**	**36**	**125**	**123**	**118**	**24**	**32**	**33**
	Ferroptosis	**23**	**27**	**-**	**34**	**128**	**128**	**124**	**26**	**34**	**33**	**27**	**35**	**-**	**41**	**90**	**118**	**117**	**48**	**56**	**50**
DHA	Native	**14**	**18**	**-**	**22**	**128**	**136**	**130**	**12**	**20**	**18**	**23**	**27**	**-**	**31**	**121**	**119**	**120**	**20**	**25**	**25**
	Ferroptosis	**31**	**33**	**-**	**34**	**118**	**125**	**113**	**41**	**32**	**38**	**33**	**38**	**-**	**36**	**77**	**114**	**110**	**45**	**51**	**60**
Non-PUFA	Native	**15**	**17**	**-**	**23**	**-**	**-**	**-**	**-**	**-**	**-**	**20**	**24**	**-**	**29**	**-**	**-**	**-**	**-**	**-**	**-**
	Ferroptosis	**19**	**25**	**-**	**28**	**-**	**-**	**-**	**-**	**-**	**-**	**22**	**26**	**-**	**30**	**-**	**-**	**-**	**-**	**-**	**-**
Cholesterol	Native			**11**										**17**							
	Ferroptosis			**18**										**21**							

Table 3 shows the average α1 angles of LA, AA, DHA, and non-PUFA phospholipids increased in both ferroptosis monolayers *versus* native. Additionally, Figure 7A shows the α1 profiles of these groups shifted to higher angles in both ferroptosis monolayers. Similarly, average α2 angles increased for these groups in both ferroptosis monolayers (Table 3). The α2 profiles also shifted to higher angles (Figure 7B). The α1 and α2 increases were more significant for hydroperoxidized PUFA *versus* non-PUFA phospholipids, indicating more interfacial orientation in ferroptosis. Increased α1 and α2 correspond to greater area per lipid and decreased the thickness of the ferroptosis membrane. Moreover, Average β angles increased for all groups in both ferroptosis monolayers (Table 3). Figure 7C shows β profiles shifted to higher angles, with more significant shifts for hydroperoxidized PUFA phospholipids. The enhancement in β indicates an increase in the angle between sn1 and sn2 chains, also increasing area per lipid and decreasing thickness.

In both monolayers of the ferroptosis membrane, average angles of γ1, γ2_1, and γ2_2 decreased for LAs, AAs, and DHAs compared to the native membrane (Table 3). Similarly, Figures 7D–F show the decreased γ1, γ2_1, and γ2_2 angles for these groups in the ferroptosis membrane. The lower γ1, γ2_1, and γ2_2 angles indicate more significant bending at oxidation sites and shorter chain lengths. These results suggest a greater increase in bend angles at oxidation sites for the ferroptosis membrane phospholipids *versus* the native membrane. In addition, LAs, AAs, and DHAs showed increased average angles of δ1, δ2_1, and δ2_2 in both ferroptosis monolayers (Table 3). Figures 7G–I demonstrate shifts to higher angles for δ1, δ2_1, and δ2_2 in both monolayers, indicating pulling of oxidized carbons (OOH moiety) towards the lipid-water interface, resulting in a decrease in chain length on the *z*-axis. These findings suggest increased bending to polar interface at oxidation sites in ferroptosis phospholipids. Notably, the outer monolayer angles of δ1, δ2_1, and δ2_2 were higher at the oxidation site of tails, indicating a greater outer monolayer impact in the ferroptosis conditions compared to the inner layer.

Significant geometry differences were observed between the monolayers in the native and ferroptosis membranes. In both membranes, α1, α2, β, δ1, δ2_1, and δ2_2 were higher in the outer monolayer, indicating it is thinner than the inner layer. This was supported by the analysis of the APL and monolayer thickness confirming a larger APL and thinner thickness in the outer monolayers (Supplementary Table S13). These variations are likely attributable to the lipid composition, with the outer layer containing 143 lipids compared to 157 in the inner layer. The outer layer is predominantly composed of PC and SM, while the inner layer consists mainly of PE. The hydrophobic -N(CH_3_)^3+^ groups in PC and SMs lipids form clathrate-like structures, allowing hydrogen bonding with water molecules and thereby extending the APL. In contrast, PE lipids directly hydrogen bond with water, condensing their head group APL and increasing the monolayer thickness (Petrache et al., 2000).

The significant structural changes observed in ferroptosis conditions result in weaker lipid packing and create more space for hydroperoxidized long-tail PUFAs to penetrate the weakened outer monolayer interface. These oxidation-induced geometry changes agree with findings from previous studies (Boonnoy et al., 2015; Siani et al., 2016).

### Hydrogen bond analysis

The changes in bilayer structure and water permeability are linked to the propensity of hydroperoxidized lipid tails to bend towards the water interface and form hydrogen bonds with polar groups (Garrec et al., 2014). To further investigate, we calculated the hydrogen bonds between individual phospholipids and polar interface groups. Table 4 presents the average number of hydrogen bonds formed per unit time between polar groups and various isomers of hydroperoxidized tails for each OOH. The results show that head groups such as serine and ethanolamine formed the fewest hydrogen bonds. Hydroperoxidized tails tended to bond strongly with water, and carbonyl and phosphates groups, which is consistent with findings from previous studies on simple oxidized bilayers (Wong-Ekkabut et al., 2007; Garrec et al., 2014; Oliveira et al., 2021).

**TABLE 4:** The average number of hydrogen bonds between OOH groups at various positions on the phospholipids and the polar interface components in the ferroptosis membrane per time frame (over the last 400 ns (n = 2)).

Ferroptosis membrane	OOH groups in different positions	Polar interface components					
		Total	Carbonyl	PO4	Ethanol	Serine	Water
Inner layer	sn1-LA13	**0.1118**	**0.0788**	**0.0273**	**0.0013**	**0.0044**	**0.2324**
	sn2-LA9	**0.2061**	**0.2075**	**0.0678**	**0.0042**	**0.0079**	**0.7125**
	sn2-LA13	**0.3801**	**0.1349**	**0.0395**	**0.0004**	**0.0053**	**0.3884**
	sn1-AA15	**0.1293**	**0.0954**	**0.0295**	**0.0013**	**0.0024**	**0.3577**
	sn2-AA12	**0.3143**	**0.2554**	**0.0450**	**0.0024**	**0.0037**	**0.5784**
	sn2-AA15	**0.2114**	**0.1552**	**0.0374**	**0.0018**	**0.0046**	**0.4214**
	sn1-DHA17	**0.1558**	**0.1151**	**0.0299**	**0.0014**	**0.0037**	**0.4018**
	sn2-DHA14	**0.2223**	**0.1698**	**0.0433**	**0.0027**	**0.0033**	**0.4725**
	sn2-DHA17	**0.3003**	**0.0885**	**0.0146**	**0.0005**	**0.0041**	**0.2864**
**Outer layer**	sn1- LA13	**0.2519**	**0.1359**	**0.0568**	**0.0008**	**0.0012**	**0.7238**
	sn2-LA9	**0.3192**	**0.1550**	**0.0676**	**0.0011**	**0.0005**	**0.8904**
	sn2-LA13	**0.4743**	**0.1635**	**0.0484**	**0.0009**	**0.0002**	**0.5921**
	sn1-AA15	**0.2924**	**0.1756**	**0.0576**	**0.0031**	**0.0000**	**0.7412**
	sn2-AA12	**0.3939**	**0.2028**	**0.0655**	**0.0018**	**0.0011**	**0.8398**
	sn2-AA15	**0.2583**	**0.1370**	**0.0364**	**0.0014**	**0.0008**	**0.6532**
	sn1-DHA17	**0.4620**	**0.1013**	**0.0295**	**0.0023**	**0.0000**	**0.3758**
	sn2-DHA14	**0.2967**	**0.1280**	**0.0584**	**0.0009**	**0.0002**	**0.8020**
	sn2-DHA17	**0.3380**	**0.1279**	**0.1127**	**0.0027**	**0.0001**	**0.8541**

In the inner layer, sn2-LA13 (0.3801 H-bond per time), sn2-AA12 (0.3143 H-bond per time), and sn2-DHA17 (0.3003 H-bond per time) showed stronger bonding compared to the sn2-LA9 (0.2061 H-bond per time), sn1-AA15 (0.1293 H-bond per time) and sn1-DHA17 (0.1558 H-bond per time). Likewise, in the outer layer, sn2-LA13 (0.4743 H-bond per time), sn2-AA12 (0.3939 H-bond per time), and sn1-DHA17 (0.4620 H-bond per time) displayed higher bonding than sn1-LA13 (0.2519 H-bond per time), sn2-AA15 (0.2583 H-bond per time), and sn2-DHA14 (0.2967 H-bond per time). These findings indicate that there is no consistent correlation between the types of isomers and bond strength. Furthermore, the average total number of hydrogen bonds per OOH group is not affected by the peroxide position or the type of fatty acids. Instead, the order parameters and geometry have a greater influence in determining the bond strength.

The outer layer exhibits higher α and δ angles, as detailed in Table 3, Alternatively, this can explain the strong bonding between the OOH and polar groups compared to the inner layer. Furthermore, the presence of more OOH-water hydrogen bonds in the outer layer suggests that water can penetrate this layer more easily (Figure 3). Our findings on geometry and area per lipid provide additional support, indicating that the outer layer has a more extended structure, leading to increased water permeability. Although our study primarily focused on water, it is reasonable to expect similar trends for other small polar molecules. The increased passage of these molecules could disrupt the balance of substances within cells, potentially leading to cell death.

To further illustrate this relationship, we presented the geometry (Supplementary Table S14, S15) and S_CD_ (Table 1) of phospholipids that exhibited significant differences in hydrogen bond strength. Table 5 demonstrates significant differences in the hydrogen bond strength between certain phospholipids. In the inner layer, for instance, SLPS13 had a much higher number of hydrogen bonds (0.373) compared to PLPE13 (0.009). Additionally, SLPS13 exhibited higher α2 (36°) and δ2-2 (54°) values compared to PLPE13 (α2 (31°) and δ2-2 (33°)). The S_CD_ of LA in SLPS13 (0.06) was also significantly lower *versus* PLPE13 (0.12). In the outer layer, SLPC13 demonstrated an ∼4x increase (0.704 H-bond per time) *versus* OLPC13 (0.172 H-bond per time), along with higher α2 (43°) and δ2-2 (58°) values compared to OLPC13 with α2 (31°) and δ2-2 (34°) values. Also, LA in SLPC13 (0.06) exhibited a lower S_CD_ value than OLPC13 (0.10). In the inner layer, SDPS17 exhibited a significantly higher number of hydrogen bonds (0.374 H-bond per time) compared to ODPE17 (0.034 H-bond per time). SDPS17 also displayed larger α2 (47°) and δ2-2 (44°) values compared to ODPE17 with α2 (30°) and δ2-1 (34°) values. The S_CD_ of DHAs in SDPS17 (0.05) was lower than in ODPE17 (0.10).

**TABLE 5:** The average number of hydrogen bonds between the OOH groups of phospholipids and the polar interface in the ferroptosis membrane per time frame (over the last 400 ns (n = 2)).

	Polar interface	
	**inner layer**	**outer layer**
PLPC9	**0.310**	**0.287**
PLPC13	**0.153**	**0.310**
OLPC9	**0.289**	**0.392**
OLPC13	**0.262**	**0.172**
SLPC9	**0.293**	**0.419**
SLPC13	**0.139**	**0.704**
SLPS9	**0.339**	**-**
SLPS13	**0.373**	**-**
SLPE9	**0.304**	**0.276**
SLPE13	**0.146**	**0.261**
DLPC9,13	**0.220**	**0.273**
PLPE9	**0.322**	**-**
PLPE13	**0.009**	**-**
DLPE9,13	**0.188**	**-**
SAPE12	**0.272**	**0.357**
SAPE15	**0.209**	**0.335**
SAPC12	**0.358**	**0.348**
SAPC15	**0.088**	**0.199**
SAPS12	**0.356**	**0.373**
SAPS15	**0.234**	**0.301**
DAPE12,15	**0.222**	**0.294**
DAPS12,15	**0.199**	**-**
DAPC12,15	**-**	**0.347**
SDPC14	**-**	**0.255**
SDPC17	**-**	**0.303**
SDPE14	**0.193**	**0.300**
SDPE17	**0.186**	**0.386**
ODPE14	**0.307**	**-**
ODPE17	**0.034**	**-**
SDPS14	**0.243**	**-**
SDPS17	**0.374**	**-**
DDPE14,17	**0.173**	**0.216**
DDPS14,17	**0.188**	**-**
LNSM9	**-**	**0.261**
LNSM13	**-**	**0.235**
DSM14	**-**	**0.363**
DSM17	**-**	**0.324**

These results demonstrate a correlation between hydroperoxide tail bonding and lipid geometry and order. Specifically, higher values of α and δ angles, as well as increased disorder, contribute to stronger bonding. These findings are consistent with previous studies, which have attributed robust peroxide-polar bonding to the positioning of lipids at the lipid-water interface (Wong-Ekkabut et al., 2007; Jarerattanachat et al., 2013).

### Lipids lateral mobility

**FIGURE 8:**
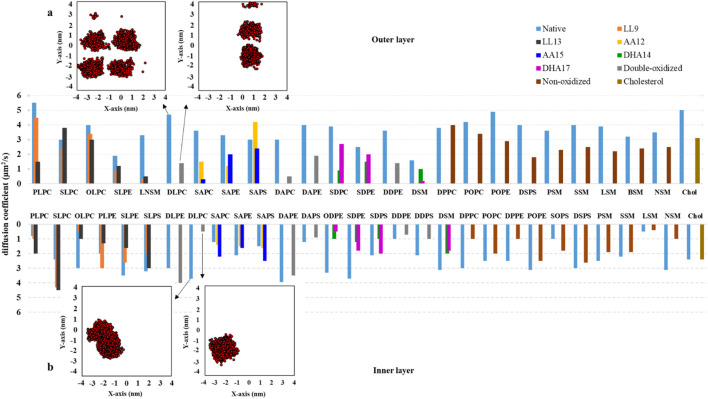
The long-term diffusion coefficients (D, measured in μm^2^.s^-1^) for lipids located in both the inner and outer monolayers of the native and ferroptosis membranes. These coefficients were calculated over the final 400 ns of 500 ns period (n = 2). **(A, B)** Depict the center of mass motion of DLPC, which was projected onto the membrane’s XY plane. This motion was tracked from 100 ns to 500 ns, with observations taken at 100 ps intervals.

Lipid peroxidation has a significant impact on various structural properties and the lateral diffusion coefficient (D) of lipids (Yeagle, 2016). To investigate this, we obtained D values for all of the lipids in both layers of native and ferroptosis membranes. Figure 8 and Supplementary Table S18 show the average D values of 33 different types of lipids in the native and oxidized membrane. In the native membrane, the D values for lipids ranged from 0.5 to 4.4 μm^2^.s^−1^ for the inner layer, and from 1.6 to 5.5 μm^2^.s^−1^ in the outer layer. In the ferroptosis membrane, the ranges were 0.4–4.5 μm^2^.s^−1^ for the inner layer and 0.2–4.5 μm^2^.s^−1^ for the outer layer. These values align with previous simulations of pure bilayers, such as PLPC (5.2 μm^2^.s^−1^) and DMPC (7.9 μm^2^.s^−1^) (Wong-Ekkabut et al., 2007; Wohlert and Edholm, 2006), as well as fluorescence photobleaching measurements in fibroblasts (1 μm^2^.s^−1^), eggs (2 μm^2^.s^−1^), and erythrocytes (0.8–1.8 μm^2^.s^−1^ for the inner layer, 0.14–0.35 μm^2^.s^−1^ for the outer layer) (Yeagle, 2016; Morrot et al., 1986; Cribier et al., 1990).

The average D values for lipids in the inner/outer layers of the native and ferroptosis membranes were 2.5/3.6 μm^2^.s^−1^ and 1.9/1.9. It can be observed that, on average, the oxidized membrane’s lipids diffuse more slowly in both layers compared to the native membrane, with a greater reduction in diffusion observed in the outer layer than in the inner layer.

In the oxidized inner layer, phospholipids with certain tails, such as AA15 show increased average D of approximately 19%. On the other hand, phospholipids including LA9, LA13, AA12, DHA14, DHA17, non-PUFA, and double-PUFA chains (double-LA, -AA, -DHA) show decreased average D values by approximately 17%, 17%, 0.06%, 57%, 50%, 31%, and 14% respectively. Also, In the oxidized inner layer, the average D value of most lipid types was decreased except DLPE9,13, PLPC13, SLPC9, SLPC13, SAPC12, SAPC15, SAPS12, SAPS15 and SOPS were increased with average values exceeding ∼1.06 μm^2^.s^−1^.

In the oxidized outer layer, phospholipids containing LA9, LA13, AA12, AA15, DHA14, DHA17, non-PUFA, and double-PUFA chains displayed decreased average D values of approximately 34%, 43%, 30%, 52%, 62%, 54%, 40%, and 66%, respectively. Generally, most lipids in the outer layer diffused more slowly in the ferroptosis membrane. However, specific lipids like SLPC13 and SAPS12 exhibited faster diffusion, with average values exceeding ∼1.1 μm^2^.s^−1^ (Figure 8). Additionally, cholesterol also demonstrated slower diffusion in the oxidized outer layer compared to the native membrane.

Previous studies similarly found slower diffusion in oxidized POPC compared to pure non-oxidized POPC bilayers (Wiczew et al., 2021; Guo et al., 2016). In our study, we observed an increased area per lipid in the oxidized layers (Supplementary Table S13). With an equal number of lipid species, this results in a higher free area per lipid in the oxidized membrane. Changes in lipid geometry and order parameter values show an increased lipid sectional area in the oxidized membrane, while the area ratio ( $\frac{a_{l}}{a_{f}}$ ) remained relatively constant. Therefore, the changes in the D value of lipids during oxidation appear to primarily modify the diffusion by affecting the activation energy barrier. The high standard deviations in D values for lipids of the same type indicate that interactions with neighboring lipids significantly influence individual diffusion rates, surpassing the impact of lipid type (Supplementary Table S18). Due to the complexity of simulated systems, each lipid molecule experiences a specific Brownian path and interacts with different lipids, leading to various diffusion behaviors even among lipid molecules of the same type. Furthermore, structural modifications in the oxidized outer layer position the OOH group near interface polar groups. This enhances hydrogen bonding, increasing the activation energy and slowing the diffusion of oxidized lipids.

Generally, the study indicates that lipids move slowly on average in ferroptosis membranes. This reduced movement can affect the membrane in various ways, including the following: 1- Protein function: Slower lipid movement can reduce the speed of protein complex formation, potentially affecting the membrane’s overall function (Gohrbandt et al., 2022), 2- Lipid modification and microdomain formation: The rate of forming disordered and ordered microdomains could be affected by reduced lipid movement (Gohrbandt et al., 2022), 3- Ion homeostasis and energy conservation: Reduced lipid movement can hinder respiration due to decreased ubiquinone diffusivity, potentially affecting efficient ion balance and energy conservation (Gohrbandt et al., 2022), 4- Cell morphogenesis and disruption: Low membrane fluidity can cause significant disturbances in cell shape formation, potentially leading to cell disruption, 5- Membrane-cytoskeleton adhesion: Reduced lipid movement can increase the adhesion energy between the membrane and the cytoskeleton, affecting the cell’s mechanical properties (Sun et al., 2007), 6- Cell stiffness: Reduced movement of both lipid and protein components could increase cell stiffness, potentially disrupting normal cell function (Goose and Sansom, 2013), 7- Receptor-ligand signaling: Studies have shown that low membrane fluidity can impair signaling, likely because lateral diffusion and localization to membrane microdomains are important for ligand binding and signaling (Kwik et al., 2003; Santos and Preta, 2018).

### PMF profiles

**FIGURE 9:**
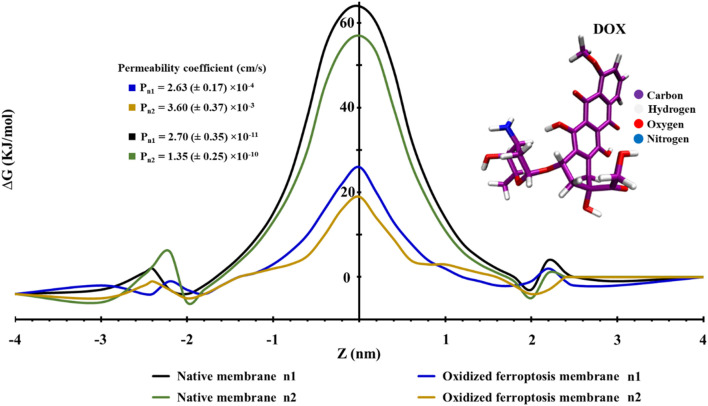
This figure illustrates the profiles of the potential of mean force (PMF) along the native and ferroptosis membranes (n = 2). The PMF profiles vary as the center of mass distance changes from the middle of the lipid bilayer for doxorubicin (DOX).

Membrane uptake is influenced by the structural changes that occur during ferroptosis. We utilized the potential of mean force (PMF) profiles to quantify this effect for DOX. As illustrated in Figure 9, DOX can easily penetrate the membranes from the water phase to the region of lipid head groups, but it encounters a significant barrier in the middle of the membranes. Based on our simulations, the ΔG_PB_ of DOX across the native (n1, n2) and ferroptosis (n1, n2) membranes was calculated to be approximately 64, 57 kJ.mol^−1^ and 26, 19 kJ.mol^−1^, respectively. This decrease is attributed to the interaction of DOX with the OOH groups in the oxidized lipid tails (Figure 9). Cell membranes act as significant energy barriers between the cytoplasm and the extracellular environment, particularly for charged and polar molecules. For example, the DMPC bilayer presents substantial free energy barriers for Na⁺ (92 kJ.mol^−1^) and Cl⁻ (100 kJ.mol^−1^) ions (Khavrutskii et al., 2009). Similarly, the POPC bilayer has energy barriers of 68 kJ.mol^−1^ for Na^+^ and 90 kJ.mol^−1^ for Cl^−^ ions. As the proportion of oxidized lipids increases such as with 50% PoxnoPC lipid in POPC bilayer-these barriers decrease by approximately 33 kJ.mol^−1^ for Na^+^ and 28 kJ.mol^−1^ for Cl-ions (Wiczew et al., 2020). Polar molecules like doxorubicin (∆G_PB_, 18.4 kJ.mol^−1^) and atenolol (∆G_PB_, 10.8 kJ.mol^−1^) form strong interactions with water and polar head groups in the DPPC bilayer, resulting in higher energy barriers. Conversely, hydrophobic molecules like ibuprofen (∆G_PB_, 6.4 kJ.mol^−1^) prefer the hydrophobic core of the bilayer (Meng and Xu, 2013). Membrane composition also plays a critical role in modulating the free energy barrier, the free energy barrier, as demonstrated with cisplatin: a model membrane of a normal cell without cholesterol shows a ∆G_PB_ of 60 kJ.mol^−1^), while a similar membrane with 33% cholesterol increases this barrier to 70 kJ.mol^−1^. Meanwhile, a DOPC bilayer exhibits a lower barrier (∆G_PB,_ 40 kJ.mol^−1^), revealing a 20–30 kJ.mol^−1^ difference depending on membrane composition (Rivel et al., 2019). Notably, the average free energy barrier of 38 kJ.mol^−1^ observed in our simulations of both normal and ferroptosis-driven oxidized membranes aligns well with these values. Specifically, for both umbrella samples of the oxidized ferroptosis membrane, the permeability coefficients were P_n1_ = 2.63 ± 0.17 × 10^−4^ cm.s^−1^/P_n2_ = 3.60 ± 0.37 × 10^−3^ cm.s^−1^, compared to P_n1_ = 2.70 ± 0.35 × 10^−11^ cm.s^−1^/P_n2_ = 1.35 ± 0.25 × 10^−10^ cm.s^−1^ for the native membrane. Consequently, the permeability coefficient of the oxidized ferroptosis membrane to DOX increases by approximately seven orders of magnitude.

The permeability of DOX varies significantly depending on membrane composition. One study reported a DOX permeability of approximately 9.0 ± 0.74 × 10^−8^ cm.s^−1^ for HepG2 cancer cells (Degerstedt et al., 2023), while another found a permeability of 6.72 × 10⁻⁷ cm.s⁻^1^ through human intestinal cells (Lee et al., 2023). In contrast, DOX permeability in Doxil liposomes was notably lower, ranging from 1 to 3 × 10⁻^12^ cm.s^−1^ (Russell et al., 2018). Computational studies on cisplatin showed similar variability: permeability in a normal cell model membrane with 33% cholesterol was 1.74 ± 0.03 × 10^−10^ cm.s^−1^, in a cancer cell model membrane it was 1.59 ± 0.06 × 10^−11^ cm.s^−1^, and in a DOPC bilayer it reached 2.2 ± 0.05 × 10^−5^ cm.s^−1^, spanning 5 to 6 orders of magnitude (Rivel et al., 2019). The permeability results from our modeled membranes align with these findings, though variations arise due to differences in evaluation methods and lipid compositions.

Our findings demonstrate significant increases in membrane fluidity, disorder, and permeability in oxidized membranes, suggesting a potential feedback loop between ferroptosis inducers and ROS accumulation within cancer cells, which may enhance therapeutic efficacy. Cancer cells, compared to normal cells, have higher levels of iron and ROS, making them more susceptible to ferroptosis (Hajipour Verdom et al., 2018; Hassannia et al., 2019). However, the high PUFA and iron content in non-cancerous cells such as neurons and cardiomyocytes, coupled with their lower levels of protective enzymes, presents a challenge; these cells are also vulnerable to ROS-induced lipid peroxidation (Jian et al., 2022; Fang et al., 2019). This susceptibility highlights the utility of these cells as models for assessing oxidative stress impacts on membrane integrity and permeability.

Notably, our results show that ferroptosis significantly increases membrane permeability to DOX. As ROS promotes lipid peroxidation, membrane permeability increases, facilitating DOX uptake and compounding oxidative damage, particularly in cardiac tissues, which constrains the clinical applicability of ferroptosis-targeted therapies. DOX exerts its effects by inhibiting topoisomerase II, a key enzyme for DNA replication and repair, thus inducing apoptosis in rapidly dividing cells (Walker and Nitiss, 2002). However, its cardiotoxic effects are increasingly attributed to excess ROS, which promotes lipid peroxidation in cell membranes (Nitiss, 2009; Pogorelcnik et al., 2013; Cvijetić et al., 2023; Li et al., 2022; Tai et al., 2023). Our oxidized membrane model offers insights into potential modifications to DOX or its delivery methods to mitigate off-target oxidative damage by minimizing unintended DOX uptake in non-target tissues.

## Conclusion

This study utilized extensive MD simulations to investigate the molecular consequences of lipid oxidation on realistic, protein-free membrane domains under ferroptosis conditions. The key findings of this study reveal several important insights. First, no significant correlation was observed between varying ratios of oxidized PUFA-phospholipid isomers and changes in their structural configuration or dynamics. Secondly, lipid oxidation leads to increased disorder in the membrane, affecting its overall organization. Thirdly, the oxidation process alters the geometry of lipids, resulting in changes to their shape and arrangement. Fourthly, Oxidation impacts the dynamics of lipid diffusion, altering their movement within the lipid bilayer. Lastly, the permeability of the oxidized membrane domain increases due to ferroptosis, potentially facilitating the passage of DOX across the membrane. These molecular insights improve our understanding of how ferroptosis-induced structural and dynamic changes compromise cell membrane integrity, a mechanism that could be leveraged in chemotherapy. Our results offer a molecular framework for understanding ROS-mediated cardiotoxicity in DOX-treated cells and could inform strategies to reduce oxidative damage in cardiac tissues, such as antioxidants co-treatments or the design of DOX analogs with reduced ROS-inducing potential. This perspective will be valuable for those investigating the molecular mechanisms underlying chemotherapy side effects and the therapeutic implications of DOX usage. Although there are limitations to this study, including limited information about the real components of human cell membranes, a lack of topological parameters for hydroperoxydized phospholipids, and hardware constraints, it represents a significant advancement in our knowledge of the mechanisms underlying ferroptosis. Future studies can build on these discoveries to explore potential therapeutic targets for inducing ferroptosis in cancer and inhibiting ferroptosis in neurodegenerative disorders. Overall, the multi-scale simulations provide unprecedented molecular-level resolution of the effects of lipid oxidation on cell membranes, shedding new light on the complex biophysical processes involved in ferroptosis.

## Data Availability

The original contributions presented in the study are included in the article/Supplementary Material, further inquiries can be directed to the corresponding authors.

## Glossary

DPPC: 1,2-dipalmitoyl-sn-glycero-3-phosphatidylcholine (16:0/16:0)
POPC: 1-palmitoyl-2-oleoyl-sn-glycero-3-phosphatidylcholine (16:0/18:1-9Z)
PLPC: 1-palmitoyl-2-linoleoyl-sn-glycero-3-phosphatidylcholine (16:0/18:2-9Z, 12Z)
SLPC: 1-stearoyl-2-linoleoyl-sn-glycero-3-phosphatidylcholine (18:0/18:2(9Z, 12Z))
OLPC: 1-oleoyl-2-linoleoyl-sn-glycero-3-phosphatidylcholine (18:1(9Z)/18:2(9Z,12Z))
DLPC: 1,2-dilinoleoyl-sn-glycero-3-phosphatidylcholine (18:2(9Z,12Z)/18:2(9Z,12Z))
SAPC: 1-stearoyl-2-arachidonoyl-sn-glycero-3-phosphatidylcholine (18:0/20:4-Z5,8Z,11Z,14Z)
DAPC: 1, 2-diarachidonoyl-sn-glycero-3-phosphatidylcholine (20:4-Z5,8Z,11Z,14Z/20:4-Z5,8Z,11Z,14Z)
SDPC: 1-stearoyl-2-docosahexaenoyl-sn-glycero-3-phosphatidylcholine (18:0/22:6-Z4,7Z,10Z,13Z,16Z,19Z)
DPPE: 1,2-dipalmitoyl-sn-glycero-3-phosphatidylethanolamine (16:0/16:0)
POPE: 1-palmitoyl-2-oleoyl-sn-glycero-3-phosphoethanolamine (16:0/18:1)
PLPE: 1-palmitoyl-2-linoleoyl-sn-glycero-3- phosphatidylethanolamine (16:0/18:2 (9Z, 12Z))
SLPE: 1-stearoyl-2-linoleoyl-sn-glycero-3- phosphatidylethanolamine (18:0/18:2 (9Z, 12Z))
DLPE: 1,2-dilinoleoyl-sn-glycero-3- phosphatidylethanolamine (18:2 (9Z,12Z)/18:2 (9Z,12Z))
SAPE: 1-stearoyl-2-arachidonoyl-sn-glycero-3- phosphatidylethanolamine (18:0/20:4-Z5,8Z,11Z,14Z)
DAPE: 1, 2-diarachidonoyl-sn-glycero-3- phosphatidylethanolamine (20:4-Z5,8Z,11Z,14Z/20:4-Z5,8Z,11Z,14Z)
SDPE: 1-stearoyl-2-docosahexaenoyl-sn-glycero-3- phosphatidylethanolamine (18:0/22:6-Z4,7Z,10Z,13Z,16Z,19Z)
ODPE: 1-oleoyl-2-docosahexaenoyl-sn-glycero-3- phosphatidylethanolamine (18:1/22:6-Z4,7Z,10Z,13Z,16Z,19Z)
DDPE: 1.2 -didocosahexaenoyl-sn-glycero-3- phosphatidylethanolamine (22:6-Z4,7Z,10Z,13Z,16Z,19Z/22:6-Z4,7Z,10Z,13Z,16Z,19Z)
DSPS: 1,2 -distearoyl-sn-glycero-3-phosphatidylserine (18:0/18:0)
SOPS: 1-stearoyl-2-oleoyl-sn-glycero-3-phosphatidylserine (18:0/18:1)
SLPS: 1-stearoyl-2-linoleoyl-*sn*-glycero-3-phosphoserine (18:0/18:2(9Z, 12Z))
SAPS: 1-stearoyl-2-arachidonoyl-sn-glycero-3-phosphatidylserine (18:0/20:4-Z5,8Z,11Z,14Z)
DAPS: 1, 2-diarachidonoyl-sn-glycero-3-phosphatidylserine (20:4-Z5,8Z,11Z,14Z/20:4-Z5,8Z,11Z,14Z)
SDPS: 1- stearoyl-2-docosahexaenoyl-sn-glycero-3- phosphatidylserine (18:0/22:6-Z4,7Z,10Z,13Z,16Z,19Z)
DDPS: 1.2 -didocosahexaenoyl-sn-glycero-3- phosphatidylserine (22:6-Z4,7Z,10Z,13Z,16Z,19Z/22:6-Z4,7Z,10Z,13Z,16Z,19Z)
PSM: N-palmitoyl Sphingomyelin/SM (18:1/16:0)
SSM: N-stearoyl-D-erythro-Sphingosylphosphorylcholine SM (18:1/18:0)
LNSM: N- linoleoyl -D-erythro- Sphingosylphosphorylcholine SM (18:1/18:2-9Z, 12Z)
NSM: N-lignoceroyl-D-erythro- Sphingosylphosphorylcholine SM (18:1/24:0)
BSM: N-docosanoyl-D-erythro-Sphingosylphosphorylcholine SM (18:1/22:0)
LSM: N-nervonoyl-D-erythro- Sphingosylphosphorylcholine SM (18:1/24:1(15Z))
DSM: N-docosahexaenoyl-D-erythro- Sphingosylphosphorylcholine SM (18:1/22:6 (4Z,7,10,13,16,19))
