# Supplementary material for: Protein-free domains in native and ferroptosis-driven oxidized cell membranes: a molecular dynamics study of biophysical properties and doxorubicin uptake
Source: Front Mol Biosci. 2024 Nov 14;11:1494257. doi: 10.3389/fmolb.2024.1494257 (PMC11602475; doi:10.3389/fmolb.2024.1494257)
Supplement: Supplementary file 1 [file Table1.pdf]

Supplementary Figures for:

Protein-Free Domains in Native and Ferroptosis-Driven Oxidized Cell Membranes: A Molecular Dynamics Study of Biophysical Properties and Doxorubicin Uptake

Yaser Shabanpour[1], Behnam Hajipour-Verdom[1], Parviz Abdolmaleki[1,*], Mozhgan Alipour[2,*].

[1] Department of Biophysics, Faculty of Biological Sciences, Tarbiat Modares University, Tehran, 14115-154, Iran.

[2] Functional Neurosurgery Research Center, Shohada Tajrish Comprehensive Neurosurgical Center of Excellence, Shahid Beheshti University of Medical Sciences, Tehran, Iran.

***Corresponding Authors:**

1- Parviz Abdolmaleki; E-mail: parviz@modares.ac.ir

2- Mozhgan Alipour; E-mail: mozhgan.alipour@sbmu.ac.ir

Supplementary Figures and Legends

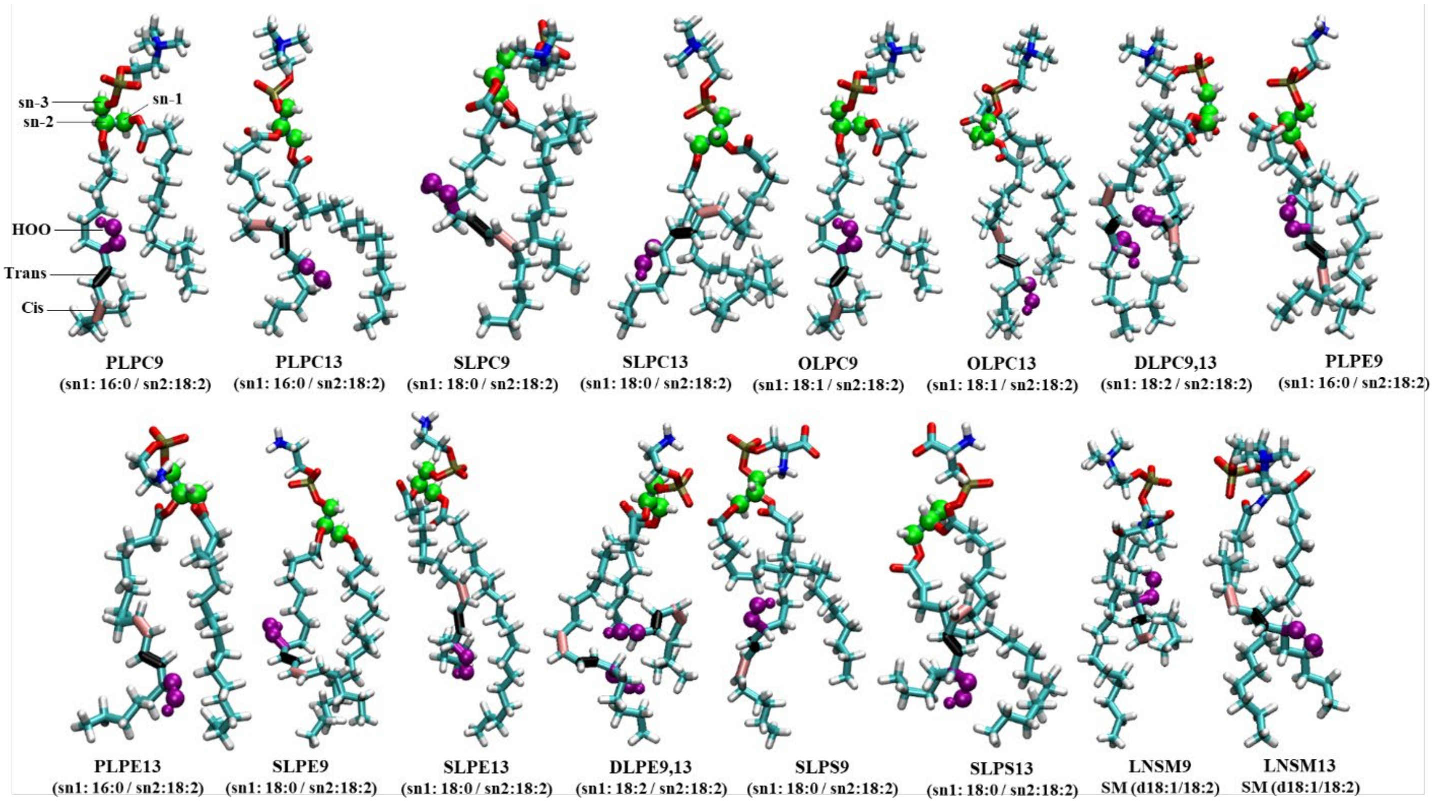

Supplementary Figure S1. We observed that the ferroptosis membrane contains phospholipids with hydroperoxidized linoleic acid (LLA). The hydroperoxidation of carbon 9 and 13 on linoleic acid leads to 9-HPODE (18:2,10E,12Z) and 13-HPODE (18:2,9Z,11E), respectively. When linoleic acid is only in the sn-2 position of a phospholipid, two isomers are formed with each of 9-HPODE and 13-HPODE. However, when both sn-1 and sn-2 positions have linoleic acid, 13-HPODE is located in the sn-1 position, and 9-HPODE is in the sn-2 position. The green balls represent glycerol carbons in sn-1, sn-2, and sn-3 positions. Purple balls represent hydroperoxidized groups (OOH), black bonds represent double bonds in trans configurations, and pink bonds represent double bonds in cis configurations. The colors of cyan, red, blue, brown, and white represent carbon, oxygen, nitrogen, sulfur, and hydrogen atoms, respectively.

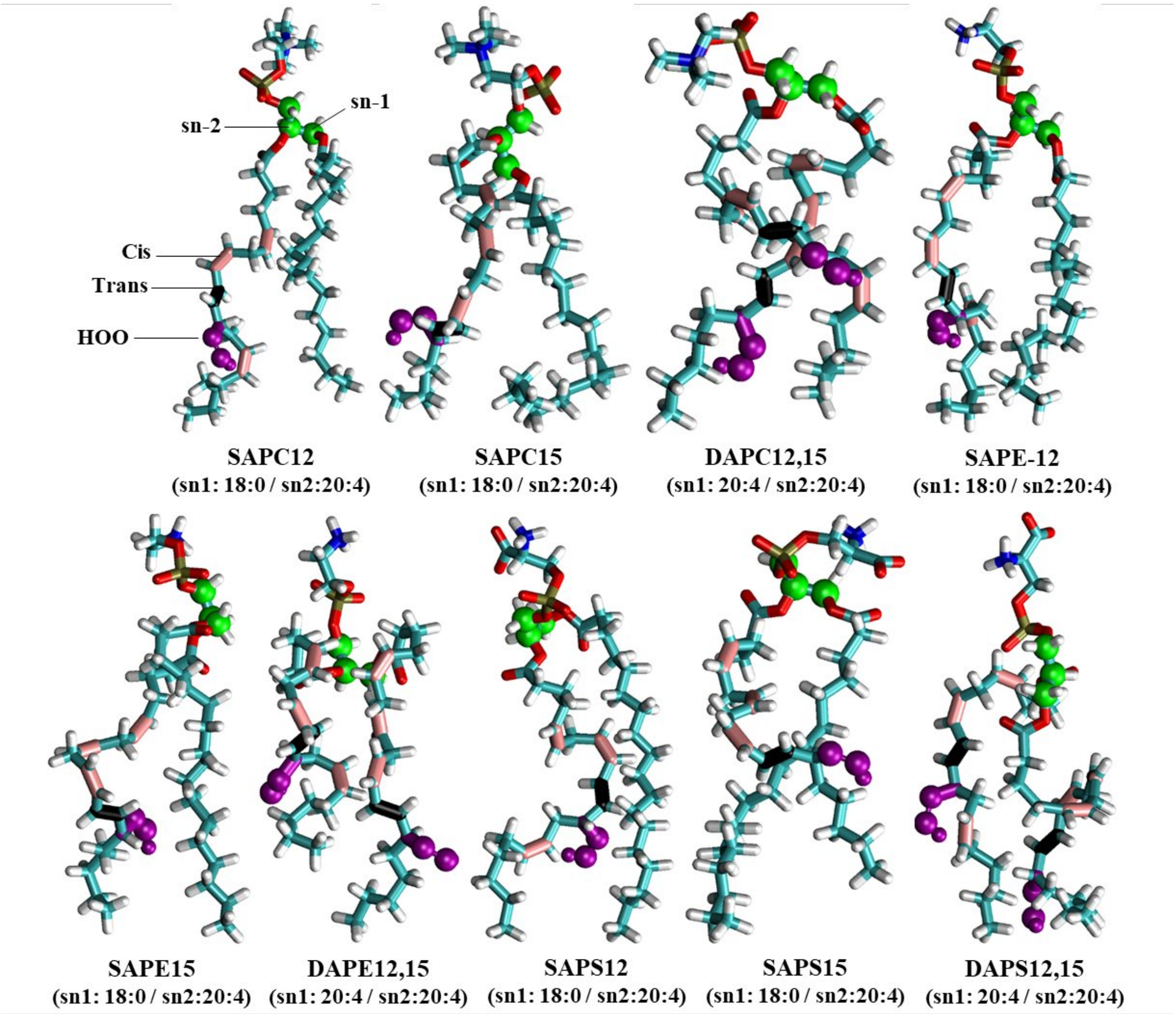

Supplementary Figure S2. We detected phospholipids in the ferroptosis membrane that contain hydroperoxidized arachidonic acid (AA). The hydroperoxidation of carbon 12 and carbon 15 on AA leads to the formation of 12-HPETE (20:4,5Z,8Z,10E,14Z) and 13-HPETE (20:4,5Z,8Z,11Z,13E), respectively. When AA is only in the sn-2 position of a phospholipid, two isomers are formed with each of 12-HPETE and 15-HPETE. However, when both sn-1 and sn-2 positions have AA, 15-HPETE is located in the sn-1 position and 12-HPETE is in the sn-2 position. The green balls represent glycerol carbons in sn-1, sn-2, and sn-3 positions, purple balls represent hydroperoxidized groups (OOH), black bonds represent double bonds in trans configurations, and pink bonds represent double bonds in cis configurations. The colors of cyan, red, blue, brown, and white represent carbon, oxygen, nitrogen, sulfur, and hydrogen atoms, respectively.

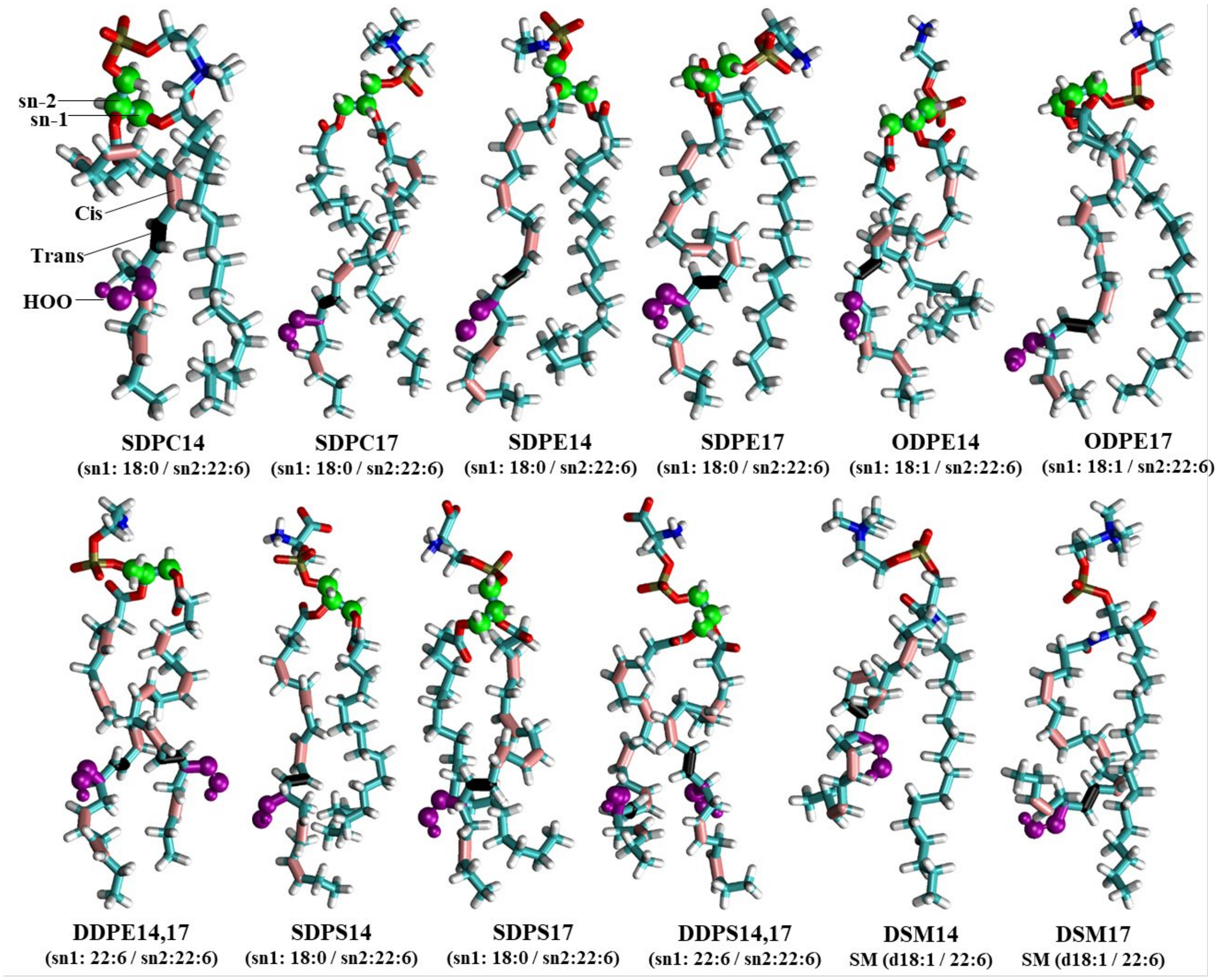

Supplementary Figure S3. We found phospholipids in the ferroptosis membrane that contain hydroperoxidized docosahexaenoic acid (DHA). The hydroperoxidation of carbon 14 and carbon 17 on DHA leads to the formation of 14-HPDHA (22:6,4Z,7Z,10Z,12E,16Z,19Z) and 17-HPDHA (22:6,4Z,7Z,10Z,13Z,15E,19Z), respectively. When DHA is only in the sn-2 position of a phospholipid, two isomers are formed with each of 14-HPDHA and 17-HPDHA. However, when both sn-1 and sn-2 positions have DHA, 17-HPDHA is located in the sn-1 position and 14-HPDHA is in the sn-2 position. The green balls represent glycerol carbons in sn-1, sn-2, and sn-3 positions, purple balls represent hydroperoxidized groups (OOH), black bonds represent double bonds in trans configurations, and pink bonds represent double bonds in cis configurations. The colors of cyan, red, blue, brown, and white represent carbon, oxygen, nitrogen, sulfur, and hydrogen atoms, respectively.

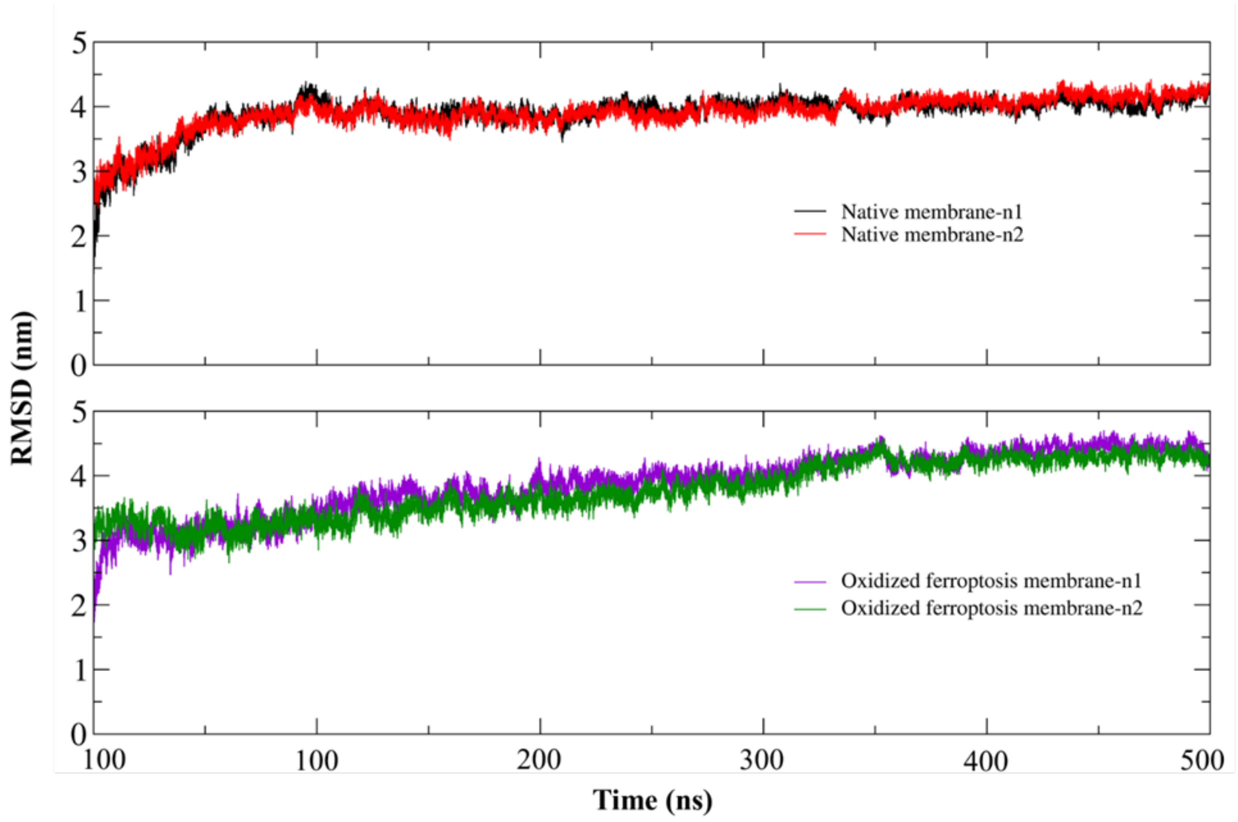

Supplementary Figure S4. The root-mean-square deviation (RMSD) of the native and ferroptosis membranes reached a steady state (n = 2). Additionally, it was observed that the n1 and n2 simulations of each membrane are similar to a high degree.

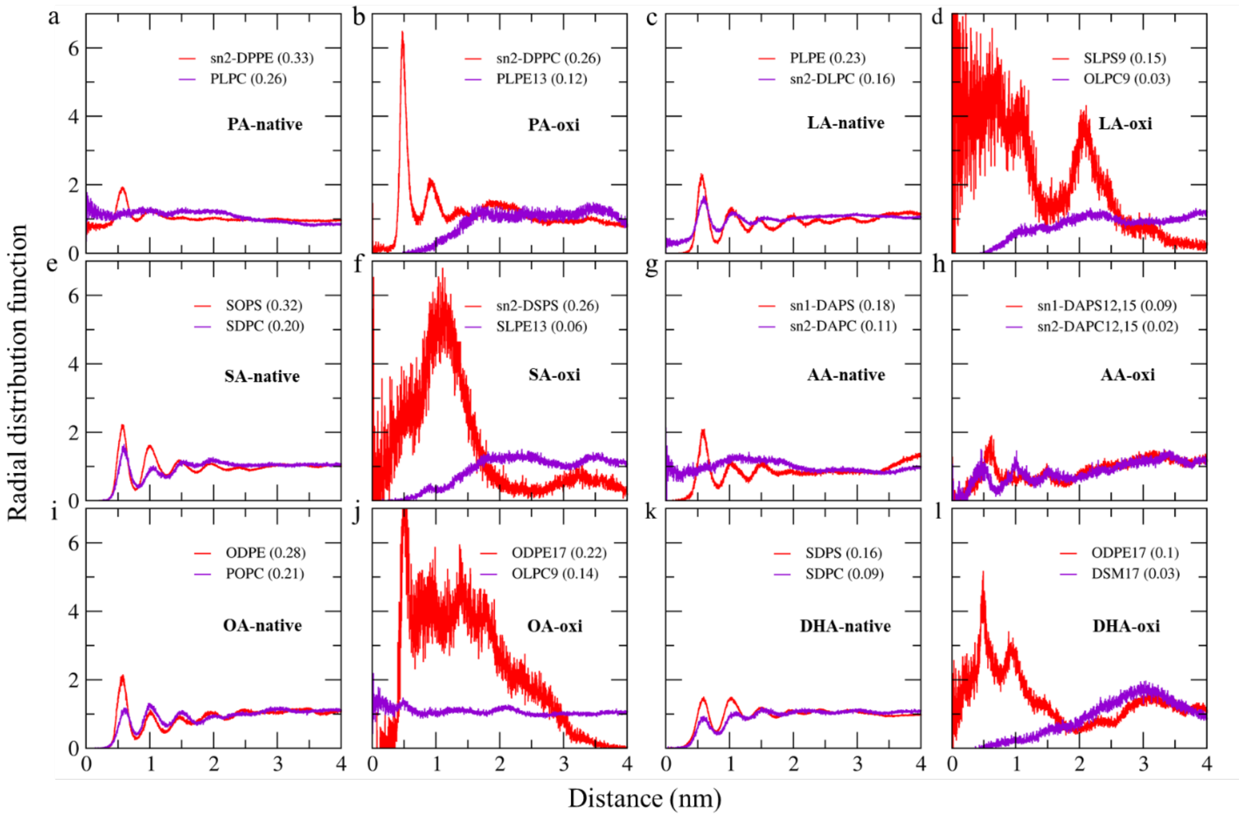

Supplementary Figure S5. The radial distribution functions (RDFs) of spatial distribution between cholesterol molecules and the carbon atoms ranging from c1-c10 of palmitic acid (PA), stearic acid (SA), oleic acid (OA), linoleic acid (LA), arachidonic acid (AA), and docosahexaenoic acid (DHA) within both native and ferroptosis membranes (n = 2). PA, SA, OA, LA, AA, and DHA exhibited varying degrees of order parameter values (S_{CD} values were shown in parentheses), with either the highest or lowest level of order. Oxi: Ferroptosis membrane.
