# Supplementary material for: Protein-free domains in native and ferroptosis-driven oxidized cell membranes: a molecular dynamics study of biophysical properties and doxorubicin uptake
Source: Front Mol Biosci. 2024 Nov 14;11:1494257. doi: 10.3389/fmolb.2024.1494257 (PMC11602475; doi:10.3389/fmolb.2024.1494257)
Supplement: Supplementary file 2 [file DataSheet1.pdf]

Supplementary Information for:

Protein-Free Domains in Native and Ferroptosis-Driven Oxidized Cell Membranes: A Molecular Dynamics Study of Biophysical Properties and Doxorubicin Uptake

Yaser Shabanpour[1], Behnam Hajipour-Verdom[1], Parviz Abdolmaleki[1,*], Mozhgan Alipour[2,*].

[1] *Department of Biophysics, Faculty of Biological Sciences, Tarbiat Modares University, Tehran, 14115-154, Iran.*

[2] *Functional Neurosurgery Research Center, Shohada Tajrish Comprehensive Neurosurgical Center of Excellence, Shahid Beheshti University of Medical Sciences, Tehran, Iran.*

***Corresponding Authors:**

1- Parviz Abdolmaleki; E-mail: parviz@modares.ac.ir

2- Mozhgan Alipour; E-mail: mozhgan.alipour@sbmu.ac.ir

Supplementary Methods

Models

The simulation boxes in the present calculations were constructed with 200 phospholipids and 100 cholesterol molecules. The specific lipid types and compositions of the native and ferroptosis membranes can be found in **Supplementary Tables S2, S3**, respectively. The ratio of cholesterol to phospholipids was set at 50% for both systems. Furthermore, the distribution of phospholipids within the membrane layers was asymmetrical, based on the experimental values [1]. In the inner membrane layer, the percentage of PC, PE, PS, and SM were 30%, 80%, 94%, and 12%, respectively. On the other hand, in the outer layer, the corresponding percentages were 70%, 20%, 6%, and 88%, respectively. To determine the fatty acid types present in each phospholipid, we referred to analytical knowledge of the human red cell plasma membrane [1] and used the information provided in **Supplementary Table S1**. The fatty acid tail components (N_{tail}) were assigned based on this reference, considering the specific fatty acid types: 16:0, 18:0, 18:1, 18:2, 20:4, 22:0, 22:6, and 24:0.

Supplementary Results and Discussion

Mass density profiles

Recent studies have utilized high-resolution X-ray techniques to determine the structural properties of erythrocyte membrane domains in two states: ordered, characterized by an all-trans configuration of lipid tails, and disordered, where the lipid chains adopt a bent configuration [2] (**Supplementary Table S13**). The determined area per lipid for the ordered and disordered domains was found to be 0.38 nm^2 and 0.50 nm^2, respectively. These values are near to the area per lipid in our native membrane (0.45 nm^2 for the inner layer and 0.50 nm^2 for the outer layer) and ferroptosis membrane (0.51 nm^2 for the inner layer and 0.56 nm^2 for the outer layer) and the difference is due to the presence of peptides within the domains. Notably, previous simulations have reported consistent findings with our observations. For example, a 15% oxidized POPC bilayer showed an increase in the area per lipid to ~0.04 nm^2 and a decrease in thickness to 0.3 nm [2]. Similarly, bilayers containing 25% PLPC-9-oxi, 50% PLPC-9-oxi, 25% PLPC-13-oxi, and 50% PLPC-13-oxi demonstrated an increase in the area per lipid to ~0.03 nm^2, 0.05 nm^2, 0.04 nm^2, and 0.06 nm^2, respectively, compared to pure PLPC. These bilayers also exhibited a decrease in thickness to 0.02 nm, 0.15 nm, 0.08 nm, and 0.29 nm, respectively [3] (**Supplementary Table S13**). These findings confirm that our all-atom force field parameters effectively capture the behavior of oxidized lipids within the membrane. The larger surface area in the ferroptosis membrane can be attributed to the bending of the hydroperoxide group toward the polar interface. Additionally, a fluorescence study supported by electron density profiles indicated that the hydroperoxide group of DOPC-OOH, DOPC-2OOH, and POPC-OOH bilayers is located near the polar interface [4].

Lipids order parameter

As **Table 1** and **Figures 4, 5** present, the identical lipid tails in different lipid species exhibit varying order parameters (S_{CD}). The S_{CD} values indicate the level of order or fluidity in the lipid tails. This variance can be attributed to the elevation in lipid order parameters when positioned near high-ordered domains and cholesterol. To investigate the reason behind this difference, we analyzed the radial distribution of cholesterol around those tails that show the highest difference in S_{CD} values. The findings reveal that in both the native and ferroptosis membranes, there is more cholesterol around the tails with higher S_{CD} values (**Supplementary Figure S5**). These findings collectively suggest that the presence of cholesterol near lipid tails can restrict their fluidity by limiting their motional freedom.

Regarding **Table 2**, the average S_{CD} of PAs, SAs, OAs, LAs, AAs, and DHAs in the sn1and sn2 were decreased in the ferroptosis membrane, indicating increased motional freedom of both oxidized and non-oxidized tails during ferroptosis conditions.

Lipid tails geometry

The α1, α2, β, δ1, δ2_1, and δ2_2 angles indicate the membrane structure and packing in detail. So we want to discuss them, completely. **Table 3** indicates that compared to the native membrane, the average angles of α1 for phospholipids containing LAs, AAs, DHAs, and non-PUFAs in the inner layer of the ferroptosis membrane increased by 2, 6, 17, and 4 degrees, respectively. In the outer layer, these angles increased by 6, 6, 1, and 2 degrees, respectively. Additionally, **Figure 5a** shows that in the inner layer of the ferroptosis membrane, the angle distributions of these phospholipid groups for α1 were broader, with peaks shifted approximately 20 degrees to higher angles, compared to the native membrane. The outer layer also exhibited broader α1 distributions but the peaks remained in the same positions.

In comparison to the native membrane, the average angles of α2 for these phospholipid groups in the inner layer of the ferroptosis membrane exhibited an increase of 18, 10, 15, and 8 degrees, respectively. Similarly, in the outer layer, these angles increased by 13, 1, 11, and 2 degrees, respectively (**Table 3**). **Figure 5b** illustrates that in the inner layer of the ferroptosis membrane, relative to the native membrane, the α2 angles of these phospholipid groups, excluding those containing non-PUFAs, displayed a broader distribution, with peaks shifted approximately 20 degrees towards higher angles. Likewise, in the outer layer, the distribution of α2 angles was also broader, with peaks shifted approximately 10, 10, and 20 degrees for those containing LAs, AAs, and DHA (excluding phospholipids containing non-PUFAs).

Compared to the native membrane, the phospholipid groups containing LAs, AAs, DHAs, and non-PUFAs in the inner layer of the ferroptosis membrane exhibited average β angles that increased by 13, 12, 12, and 5 degrees, respectively. Similarly, in the outer layer, these angles increased by 14, 5, 5, and 1 degrees, respectively. In **Figure 5c**, it can be observed that in the inner layer of the ferroptosis membrane relative to the native membrane, the β angles of these phospholipid groups displayed a broader distribution. The peaks of the profiles were shifted approximately 15 degrees towards higher angles for phospholipids containing LAs, AAs, and DHAs, while phospholipids containing non-PUFAs showed no shift of approximately 5 degrees. In the outer layer, the β distribution was also broader (excluding phospholipids containing non-PUFAs), with peaks shifted approximately 20, 10, and 10 degrees towards higher angles for phospholipids containing LAs, AAs, and DHAs, respectively. The enhanced degrees for α1, α2, and β angles indicate that lipid tails in the ferroptosis membrane are more extended than the native membrane which leads to a thicker membrane.

The average angles of δ1/ δ2_1/ δ2_2 for phospholipid groups showed an increase, including 3/ 14/ 28 degrees for LAs, 6/ 17/ 14 degrees for AAs, and 29/ 12/ 20 degrees for DHAs in the inner layer of the ferroptosis membrane, respectively, compared to the native membrane. In the outer layer, these angles increased by 24/ 34/ 25 for LAs, degrees and increased by 24/ 24/ 17 degrees for AAs, and increased by 25/ 26/ 35 degrees for DHAs, respectively. **Figure 5-g, h, i** show that in the inner layer of the ferroptosis membrane, relative to the native membrane, the δ1, δ2_1, and δ2_2 angles of phospholipid groups containing LAs, AAs, and DHAs were distributed more broadly. the peak site of all δ1,-δ2_1, and δ2_2 angles was shifted approximately 10 degrees to higher angles for LAs, AAs, and DHAs. Likewise, in the outer layer, the δ1, δ2_1, and δ2_2 angles were also distributed more broadly. The peaks of δ1 shifted approximately 10 degrees to the higher angles. The peaks of δ2_1 angles were shifted approximately 20 degrees higher for phospholipids containing LAs, and AAs and 30 degrees higher for DHAs. Also, the peak sites of δ2_2 angles were shifted approximately 20 and 30 degrees to higher angles for phospholipids containing AAs and DHAs in the ferroptosis membrane.

Additionally, it was observed that the α1, α2, and β parameters in both layers of the ferroptosis membrane were significantly larger than the native membrane. This suggests an increase in the area per lipid and a decrease in the membrane thickness. Furthermore, the δ1, δ2_1, and δ2_2 angles in phospholipids of the ferroptosis membrane were significantly larger than those in the native membrane, indicating that the carbons carrying the OOH moiety in LAs, AAs, and DHAs were drawn closer to the lipid-water interface.

Lipids lateral mobility

We calculated the average diffusion coefficient (D) and standard deviation (SD) from the data in **Supplementary Table S18** for five groups of phospholipids in each monolayer. In the inner

layer of the native membrane, the average D and SD for phospholipids containing LA, AA, DHA, double-PUFAs (double-LA, double-AA, double-DHA), and non-PUFA chains were 2.48 ± 0.98, 1.6 ± 0.45, 3.05 ± 0.68, 2.45 ± 1.28 and 2.34 ± 0.90 $\mu m^2.s^{-1}$, respectively, while in the outer layer, they were 3.54 ± 1.33, 3.3 ± 0.30, 2.66 ± 1.15, 3.82 ± 0.71 and 3.9 ± 0.48 $\mu m^2.s^{-1}$, respectively. In the inner layer of the oxidized membrane, the average D and SD for eight groups of phospholipids containing LA9, LA13, AA12, AA15, DHA14, DHA17, double-PUFAs (double-OOH), and non-PUFA chains were 2.2 ± 1.26, 2.17 ± 1.2, 1.5 ± 0.1, 2.1 ± 0.45, 1.06 ± 0.11, 1.43 ± 0.81, 1.76 ± 1.55 and 1.61 ± 0.72 $\mu m^2.s^{-1}$, respectively.

In the outer layer, they were 2.32 ± 1.73, 2 ± 1.35, 2.3 ± 1.65, 1.56 ± 1.11, 1.13 ± 0.32, 1.63 ± 1.28, 1.3 ± 0.58, and 2.63 ± 0.68 $\mu m^2.s^{-1}$, respectively indicating that the lipids diffusion decreased in both layers considerably in the outer layer of the ferroptosis membrane relative to the native membrane.

References

1. Yawata, Y., *Cell membrane: the red blood cell as a model*. 2006: John Wiley & Sons.
2. Himbert, S., et al., *The molecular structure of human red blood cell membranes from highly oriented, solid supported multi-lamellar membranes.* Scientific reports, 2017. **7**(1): p. 39661.
3. Wong-Ekkabut, J., et al., *Effect of lipid peroxidation on the properties of lipid bilayers: a molecular dynamics study.* Biophysical journal, 2007. **93**(12): p. 4225-4236.
4. Siani, P., et al., *An overview of molecular dynamics simulations of oxidized lipid systems, with a comparison of ELBA and MARTINI force fields for coarse grained lipid simulations.* Biochimica et Biophysica Acta (BBA)-Biomembranes, 2016. **1858**(10): p. 2498-2511.
