# Supplementary material for: Protein-free domains in native and ferroptosis-driven oxidized cell membranes: a molecular dynamics study of biophysical properties and doxorubicin uptake
Source: Front Mol Biosci. 2024 Nov 14;11:1494257. doi: 10.3389/fmolb.2024.1494257 (PMC11602475; doi:10.3389/fmolb.2024.1494257)
Supplement: Supplementary file 3 [file Image1.pdf]

Supplementary Tables for:

Protein-Free Domains in Native and Ferroptosis-Driven Oxidized Cell Membranes: A Molecular Dynamics Study of Biophysical Properties and Doxorubicin Uptake

Yaser Shabanpour[1], Behnam Hajipour-Verdom[1], Parviz Abdolmaleki[1,*], Mozhgan Alipour[2,*].

[1] *Department of Biophysics, Faculty of Biological Sciences, Tarbiat Modares University, Tehran, 14115-154, Iran.*

[2] *Functional Neurosurgery Research Center, Shohada Tajrish Comprehensive Neurosurgical Center of Excellence, Shahid Beheshti University of Medical Sciences, Tehran, Iran.*

***Corresponding Authors:**

1- Parviz Abdolmaleki; E-mail: parviz@modares.ac.ir

2- Mozhgan Alipour; E-mail: mozhgan.alipour@sbmu.ac.ir

Supplementary Tables and Legends

Supplementary Table S1. The composition and asymmetry of the modeled native plasma membrane based on human red cells. The N_{lip} represents the number of lipid molecules, PL represents phospholipids, and $\%N_{tail}$ represents the percentage of fatty acid tails related to the phospholipids. $N_{phospholipid}$ represents the number of phospholipids related to fatty acids. The fatty acids included in the model are stearic acid ([a]), oleic acid ([b]), linoleic acid ([c]), arachidonic acid ([d]), docosahexaenoic acid ([e]), palmitic acid ([f]), behenic acid ([g]), lignoceric acid ([h]), and dinervonoyl acid ([i]).

N_{lip}	PL			$\%N_{tail}$		$N_{phospholipid}$	
	Type and number of total	Inner	Outer			Inner	Outer
**200 PL**	PS + Pi (%20 of total PL) = 40	37 (%94)	3 (%6)	%51	18:0[a]	19	2
				%10	18:1[b]	4	-
				%3	18:2[c]	1	-
				%20	20:4[d]	7	1
				%16	22:6[e]	6	-
	PC (%27 of total PL) = 54	%30 = 16	%70 = 38	%37	16:0[f]	6	14
				%12	18:0	2	5
				%18	18:1	3	6
				%23	18:2	4	9
				%7	20:4	1	3
				%3	22:6	-	1
	PE (%30 of total PL) = 60	%80 = 48	%20 = 12	%19	16:0	10	2
				%21	18:0	10	3
				%17	18:1	8	2
				%7	18:2	3	1
				%20	20:4	10	2
				%16	22:6	7	2
	SM (%23 of total PL) = 46	%12 = 6	%88 = 40	%26	16:0	2	10
				%10	18:0	1	4
				5%	18:2	-	2
				%7	22:0[g]	-	3
				%5	22:6	-	2
				%20	24:0[h]	1	8
				%27	24:1[i]	2	11
**100 cholesterol**	(%50 of total PL) = 100	%50	%50	-	-	50	50

Supplementary Table S2. The types and quantities of phospholipids in the inner (In) and outer (Out) layers of the modeled native plasma membrane.

PE	In	Out	PC	In	Out	PS	In	Out	SM	In	Out
DPPE	2		DPPC	3	6	DSPS	3	1	PSM	2	10
POPE	14	4	POPC	4	10	SOPS	8		SSM	1	4
PLPE	2		PLPC	2	6	SLPS	2		LNSM		2
SLPE	2	2	SLPC	2	4	SAPS	12	2	NSM	1	8
DLPE	1		OLPC	2	2	DAPS	1		BSM		3
SAPE	12	2	DLPC	1	3	SDPS	10		LSM	2	11
DAPE	4	1	SAPC	2	4	DDPS	1		DSM		2
SDPE	6	2	DAPC		1						
ODPE	2		SDPC		2						
DDPE	3	1									

Supplementary Table S3. The types and quantities of phospholipids in the inner (In) and outer (Out) layers of the modeled ferroptosis membrane.

PE	In	Out	PC	In	Out	PS	In	Out	SM	In	Out
DPPE	2		DPPC	3	6	DSPS	3	1	PSM	2	10
POPE	14	4	POPC	4	10	SOPS	8		SSM	1	4
PLPE9	1		PLPC9	1	3	SLPS9	1		LNSM9		1
PLPE13	1		PLPC13	1	3	SLPS13	1		LNSM13		1
SLPE9	1	1	SLPC9	1	2	SAPS12	6	1	NSM	1	8
SLPE13	1	1	SLPC13	1	2	SAPS15	6	1	BSM		3
DLPE9,13	1		OLPC9	1	1	DAPS12,15	1		LSM	2	11
SAPE12	6	1	OLPC13	1	1	SDPS14	5		DSM14		1
SAPE15	6	1	DLPC9,13	1	3	SDPS17	5		DSM17		1
DAPE12,15	4	1	SAPC12	1	2	DDPS14,17	1				
SDPE14	3	1	SAPC15	1	2						
SDPE17	3	1	DAPC12,15		1						
ODPE14	1		SDPC14		1						
ODPE17	1		SDPC17		1						
DDPE14,17	3	1									

Supplementary Table S4. The connectivities, atom type, and charge definition of phospholipids that have oxidized isoforms of LLA were shown, including **(a)** sn2-9HPODE, **(b)** sn2-13HPODE, and **(c)** sn1-13HPODE/Sn2-9HPODE.

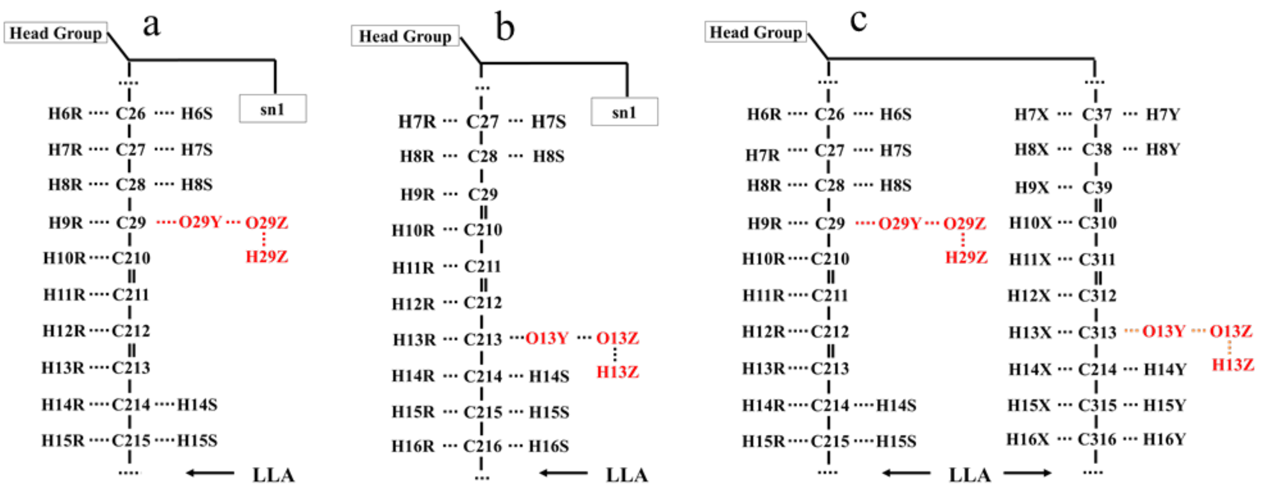

Atom	Type	Charge	Atom	Type	Charge	Atom	Type	Charge	Atom	Type	Charge
C26	CTL2	-0.18	C27	CTL2	-0.18	C26	CTL2	-0.18	C37	CTL2	-0.18
H6R	HAL2	0.09	H7R	HAL2	0.09	H6R	HAL2	0.09	H7X	HAL2	0.09
H6S	HAL2	0.09	H7S	HAL2	0.09	H6S	HAL2	0.09	H7Y	HAL2	0.09
C27	CTL2	-0.18	C28	CTL2	-0.18	C27	CTL2	-0.18	C38	CTL2	-0.18
H7R	HAL2	0.09	H8R	HAL2	0.09	H7R	HAL2	0.09	H8X	HAL2	0.09
H7S	HAL2	0.09	H8S	HAL2	0.09	H7S	HAL2	0.09	H8Y	HAL2	0.09
C28	CTL2	-0.18	C29	CG2DC1	-0.21	C28	CTL2	-0.18	C39	CG2DC1	-0.21
H8R	HAL2	0.09	H9R	HGA4	0.21	H8R	HAL2	0.09	H9X	HGA4	0.21
H8S	HAL2	0.09	C210	CG2DC1	-0.15	H8S	HAL2	0.09	C310	CG2DC1	-0.15
C29	CG311	0.08	H10R	HGA4	0.15	C29	CG311	0.08	H10X	HGA4	0.15
H9R	HGA1	0.09	C211	CG2DC2	-0.15	H9R	HGA1	0.09	C311	CG2DC2	-0.15
O29Y	OG301	-0.138	H11R	HGA4	0.15	O29Y	OG301	-0.138	H11X	HGA4	0.15
O29Z	OG301	-0.392	C212	CG2DC2	-0.15	O29Z	OG301	-0.392	C312	CG2DC2	-0.15
H29Z	HGP1	0.36	H12R	HGA4	0.15	H29Z	HGP1	0.36	H12X	HGA4	0.15
C210	CG2DC2	-0.15	C213	CG311	0.08	C210	CG2DC2	-0.15	C313	CG311	0.08
H10R	HGA4	0.15	H13R	HGA1	0.09	H10R	HGA4	0.15	H13X	HGA1	0.09
C211	CG2DC2	-0.15	O13Y	OG301	-0.138	C211	CG2DC2	-0.15	O13Y	OG301	-0.138
H11R	HGA4	0.15	O13Z	OG301	-0.392	H11R	HGA4	0.15	O13Z	OG301	-0.392
C212	CG2DC1	-0.15	H13Z	HGP1	0.36	C212	CG2DC1	-0.15	H13Z	HGP1	0.36
H12R	HGA4	0.15	C214	CTL2	-0.18	H12R	HGA4	0.15	C314	CTL2	-0.18
C213	CG2DC1	-0.21	H14R	HAL2	0.09	C213	CG2DC1	-0.21	H14X	HAL2	0.09
H13R	HGA4	0.21	H14S	HAL2	0.09	H13R	HGA4	0.21	H14Y	HAL2	0.09
C214	CTL2	-0.18	C215	CTL2	-0.18	C214	CTL2	-0.18	C315	CTL2	-0.18
H14R	HAL2	0.09	H15R	HAL2	0.09	H14R	HAL2	0.09	H15X	HAL2	0.09
H14S	HAL2	0.09	H15S	HAL2	0.09	H14S	HAL2	0.09	H15Y	HAL2	0.09
C215	CTL2	-0.18	C216	CTL2	-0.18	C215	CTL2	-0.18	C316	CTL2	-0.18
H15R	HAL2	0.09	H16R	HAL2	0.09	H15R	HAL2	0.09	H16X	HAL2	0.09
H15S	HAL2	0.09	H16S	HAL2	0.09						

Supplementary Table S5. The connectivities, atom type, and charge definition of phospholipids that have oxidized isoforms of AA were shown, including **(a)** sn2-12HPETE, **(b)** sn2-15HPETE, and **(c)** sn1-15HPETE/sn2-12HPETE.

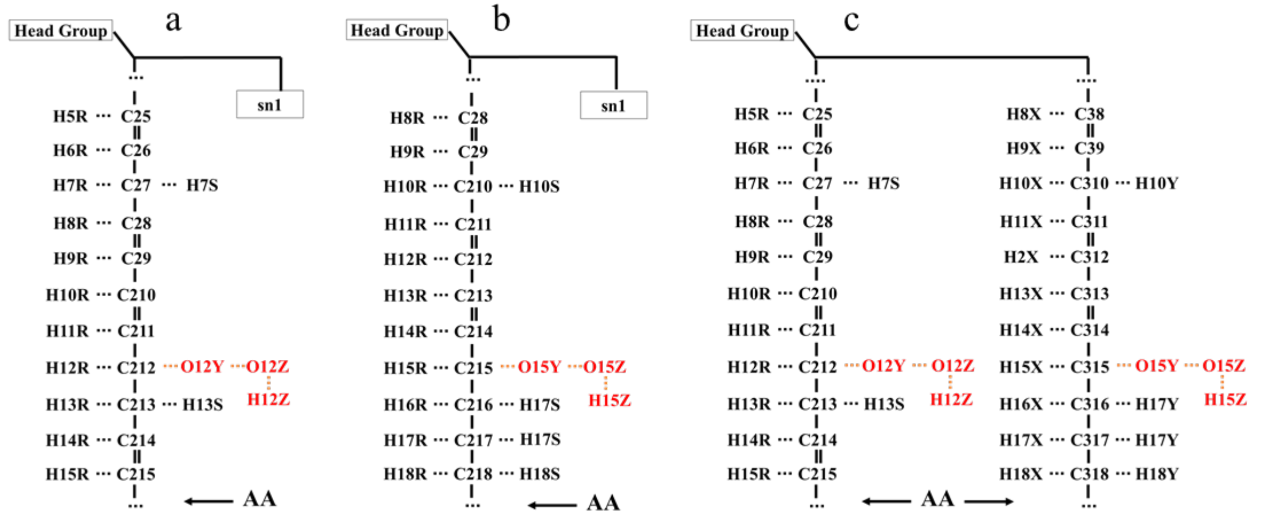

Atom	Type	Charge	Atom	Type	Charge	Atom	Type	Charge	Atom	Type	Charge
C25	CEL1	-0.15	C28	CEL1	-0.15	C25	CEL1	-0.15	C38	CEL1	-0.15
H5R	HEL1	0.15	H8R	HEL1	0.15	H5R	HEL1	0.15	H8X	HEL1	0.15
C26	CEL1	-0.15	C29	CEL1	-0.15	C26	CEL1	-0.15	C39	CEL1	-0.15
H6R	HEL1	0.15	H9R	HEL1	0.15	H6R	HEL1	0.15	H9X	HEL1	0.15
C27	CTL2	-0.18	C210	CTL2	-0.18	C27	CTL2	-0.18	C310	CTL2	-0.18
H7R	HAL2	0.09	H10R	HAL2	0.09	H7R	HAL2	0.09	H10X	HAL2	0.09
H7S	HAL2	0.09	H10S	HAL2	0.09	H7S	HAL2	0.09	H10Y	HAL2	0.09
C28	CG2DC1	-0.21	C211	CG2DC1	-0.21	C28	CG2DC1	-0.21	C311	CG2DC1	-0.21
H8R	HGA4	0.21	H11R	HGA4	0.21	H8R	HGA4	0.21	H11X	HGA4	0.21
C29	CG2DC1	-0.15	C212	CG2DC1	-0.15	C29	CG2DC1	-0.15	C312	CG2DC1	-0.15
H9R	HGA4	0.15	H12R	HGA4	0.15	H9R	HGA4	0.15	H12X	HGA4	0.15
C210	CG2DC2	-0.15	C213	CG2DC2	-0.15	C210	CG2DC2	-0.15	C313	CG2DC2	-0.15
H10R	HGA4	0.15	H13R	HGA4	0.15	H10R	HGA4	0.15	H13X	HGA4	0.15
C211	CG2DC2	-0.15	C214	CG2DC2	-0.15	C211	CG2DC2	-0.15	C314	CG2DC2	-0.15
H11R	HGA4	0.15	H14R	HGA4	0.15	H11R	HGA4	0.15	H14X	HGA4	0.15
C212	CG311	0.08	C215	CG311	0.08	C212	CG311	0.08	C315	CG311	0.08
H12R	HGA1	0.09	H15R	HGA1	0.09	H12R	HGA1	0.09	H15X	HGA1	0.09
O12Y	OG301	-0.138	O15Y	OG301	-0.138	O12Y	OG301	-0.138	O15Y	OG301	-0.138
O12Z	OG301	-0.392	O15Z	OG301	-0.392	O12Z	OG301	-0.392	O15Z	OG301	-0.392
H12Z	HGP1	0.36	H15Z	HGP1	0.36	H12Z	HGP1	0.36	H15Z	HGP1	0.36
C213	CTL2	-0.18	C216	CTL2	-0.18	C213	CTL2	-0.18	C316	CTL2	-0.18
H13R	HAL2	0.09	H16R	HAL2	0.09	H13R	HAL2	0.09	H16X	HAL2	0.09
H13S	HAL2	0.09	H16S	HAL2	0.09	H13S	HAL2	0.09	H16Y	HAL2	0.09
C214	CEL1	-0.15	C217	CTL2	-0.18	C214	CEL1	-0.15	C317	CTL2	-0.18
H14R	HEL1	0.15	H17R	HAL2	0.09	H14R	HEL1	0.15	H17X	HAL2	0.09
C215	CEL1	-0.15	H17S	HAL2	0.09	C215	CEL1	-0.15	H17Y	HAL2	0.09
H15R	HEL1	0.15	C218	CTL2	-0.18	H15R	HEL1	0.15	C318	CTL2	-0.18
			H18R	HAL2	0.09				H18X	HAL2	0.09
			H18S	HAL2	0.09				H18Y	HAL2	0.09

Supplementary Table S6. The connectivities, atom type, and charge definition of phospholipids that have oxidized isoforms of DHA were shown, including **(a)** sn2-14HPDHA, **(b)** sn2-17HPDHA and **(c)** sn1-17HPDHA/sn2-14HPDHA.

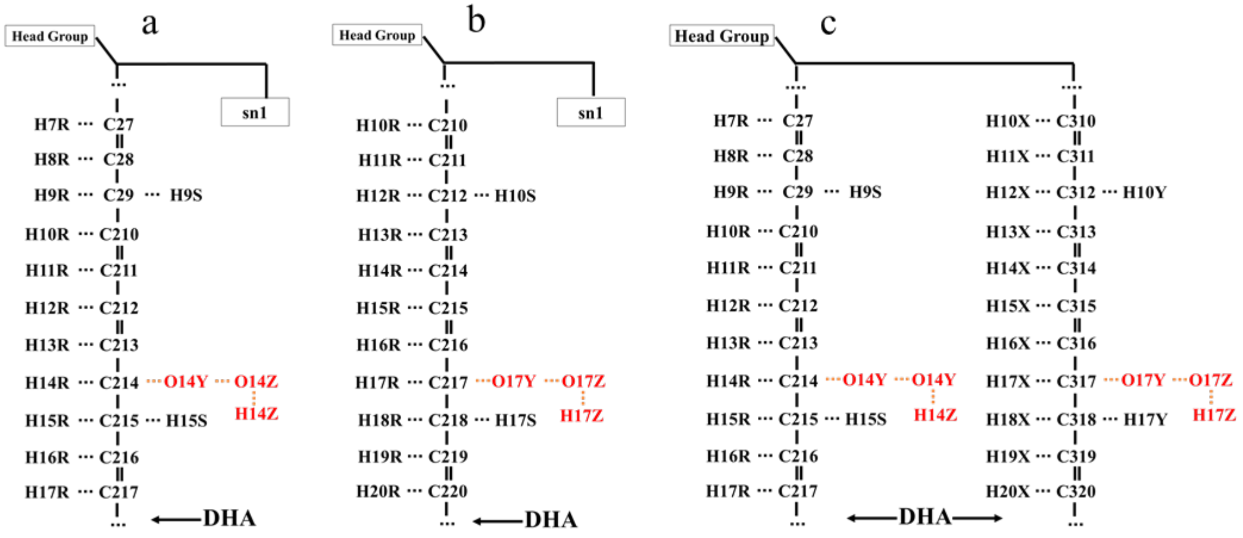

Atom name	Atom type	Charge	Atom	Type	Charge	Atom	Type	Charge	Atom	Type	Charge
C27	CEL1	-0.15	C210	CEL1	-0.15	C310	CEL1	-0.15	C27	CEL1	-0.15
H7R	HEL1	0.15	H10R	HEL1	0.15	H10X	HEL1	0.15	H7R	HEL1	0.15
C28	CEL1	-0.15	C211	CEL1	-0.15	C311	CEL1	-0.15	C28	CEL1	-0.15
H8R	HEL1	0.15	H11R	HEL1	0.15	H11X	HEL1	0.15	H8R	HEL1	0.15
C29	CTL2	-0.18	C212	CTL2	-0.18	C312	CTL2	-0.18	C29	CTL2	-0.18
H9R	HAL2	0.09	H12R	HAL2	0.09	H12X	HAL2	0.09	H9R	HAL2	0.09
H9S	HAL2	0.09	H12S	HAL2	0.09	H12Y	HAL2	0.09	H9S	HAL2	0.09
C210	CG2DC1	-0.21	C213	CG2DC1	-0.21	C313	CG2DC1	-0.21	C210	CG2DC1	-0.21
H10R	HGA4	0.21	H13R	HGA4	0.21	H13X	HGA4	0.21	H10R	HGA4	0.21
C211	CG2DC1	-0.15	C214	CG2DC1	-0.15	C314	CG2DC1	-0.15	C211	CG2DC1	-0.15
H11R	HGA4	0.15	H14R	HGA4	0.15	H14X	HGA4	0.15	H11R	HGA4	0.15
C212	CG2DC2	-0.15	C215	CG2DC2	-0.15	C315	CG2DC2	-0.15	C212	CG2DC2	-0.15
H12R	HGA4	0.15	H15R	HGA4	0.15	H15X	HGA4	0.15	H12R	HGA4	0.15
C213	CG2DC2	-0.15	C216	CG2DC2	-0.15	C316	CG2DC2	-0.15	C213	CG2DC2	-0.15
H13R	HGA4	0.15	H16R	HGA4	0.15	H16X	HGA4	0.15	H13R	HGA4	0.15
C214	CG311	0.08	C217	CG311	0.08	C317	CG311	0.08	C214	CG311	0.08
H14R	HGA1	0.09	H17R	HGA1	0.09	H17X	HGA1	0.09	H14R	HGA1	0.09
O14Y	OG301	-0.138	O17Y	OG301	-0.138	O17Y	OG301	-0.138	O14Y	OG301	-0.138
O14Z	OG301	-0.392	O17Z	OG301	-0.392	O17Z	OG301	-0.392	O14Z	OG301	-0.392
H14Z	HGP1	0.36	H17Z	HGP1	0.36	H17Z	HGP1	0.36	H14Z	HGP1	0.36
C215	CTL2	-0.18	C218	CTL2	-0.18	C318	CTL2	-0.18	C215	CTL2	-0.18
H15R	HAL2	0.09	H18R	HAL2	0.09	H18X	HAL2	0.09	H15R	HAL2	0.09
H15S	HAL2	0.09	H18S	HAL2	0.09	H18Y	HAL2	0.09	H15S	HAL2	0.09
C216	CEL1	-0.15	C219	CEL1	-0.15	C319	CEL1	-0.15	C216	CEL1	-0.15
H16R	HEL1	0.15	H19R	HEL1	0.15	H19X	HEL1	0.15	H16R	HEL1	0.15
C217	CEL1	-0.15	C220	CEL1	-0.15	C320	CEL1	-0.15	C217	CEL1	-0.15
H17R	HEL1	0.15	H20R	HEL1	0.15	H20X	HEL1	0.15	H17R	HEL1	0.15

Supplementary Table S7. The geometries and numbers of components for the inner leaflet, outer leaflet, in-solve, and out-solve of the native and ferroptosis membranes. The "in-solve" and "out-solve" represent the solvate around the inner and outer leaflets, respectively.

The characteristics of the simulation BOXs										
Membrane parts		Range			Number of molecules and ions				
		X (nm)	Y (nm)	Z (nm)	lipid	water	Sodium	Choler	Construction Approach
**Native**	Inner leaflet	0-10	0-10		157				CHARM-GUI interface
	Outer leaflet	0-10	0-10		143				
	In-solve	0-10	0-10			5230	34	12	
	Out-solve	0-10	0-10			5230	32	14	
	Box	0-10	0-10		300	10460	66	26	
**Ferroptosis**	Inner leaflet	0-10	0-10	2-4.4	157				Packmol software
	Outer leaflet	0-10	0-10	4.6-7	143				
	In-solve	0-10	0-10	0-2.3		5230	34	12	
	Out-solve	0-10	0-10	6.7-9		5230	32	14	
	Box	0-10	0-10	09	300	10460	66	26	

Supplementary Table S8. Stretching parameters for hydroperoxidized phospholipids. Units are those used in GROMACS, namely nm and kJ.mol^{-1}.nm^{-2} for distances and energies.

Atom type #1	Atom type #2	b0 [nm]	K_b [Kj.mol^{-1}.nm^{-2}]
CG311	CTL2	0.1538	186188
CG2DC2	CG311	0.1502	305432
OG301	OG301	0.14588	214553
OG301	HGP1	0.09726	364879
CG2DC1	CTL2	0.1502	305432

Supplementary Table S9. Bending parameters for hydroperoxidized phospholipids. Units are those used in GROMACS, namely nm and kJ.mol^{-1}.degree^{-2} for distances and energies.

Atom type #1	Atom type #2	Atom type #3	θ[degree]	k_θ [kJ.mol^{-1}.degree^{-2}]
CTL2	CG311	OG301	111.5	376.56
CG311	OG301	OG301	106.57	606.109
HGP1	OG301	OG301	99.56	463.163
CG2DC2	CG311	OG301	106.73	536.247
CG311	CTL2	CTL2	113.5	488.273
HAL2	CTL2	CG311	110.1	221.752
CTL2	CG311	HGA1	111.5	376.56
CG2DC2	CG311	CTL2	112.2	267.776
CG2DC2	CG311	HGA1	111.5	376.56
CG311	CG2DC2	HGA4	116	334.72
CG2DC2	CG2DC2	CG311	123.5	401.664
CG2DC1	CG2DC1	CTL2	123.5	401.664
HGA4	CG2DC1	CTL2	116	334.72
HAL2	CTL2	CG2DC1	111.5	376.56
CG2DC1	CTL2	CTL2	112.2	267.776
CEL1	CTL2	CG311	112.2	267.776
CEL1	CTL2	CG2DC1	114	251.04

Supplementary Table S10. Dihedral parameters for hydroperoxidized phospholipids. Units are those used in GROMACS, namely nm and kJ.mol^{-1} for distances and energies.

Atom type #1	Atom type #2	Atom type #3	Atom type #4	ϕ	Kϕ[KJ/mol]	Multiplicity
HGA1	CG311	CTL2	CTL2	0	1.362736	3
OG301	CG311	CTL2	CTL2	0	-3.19913	1
OG301	CG311	CTL2	CTL2	0	0.471897	2
OG301	CG311	CTL2	CTL2	0	1.677636	3
CG2DC2	CG311	CTL2	CTL2	0	-6.65949	1
CG2DC2	CG311	CTL2	CTL2	180	1.415942	2
CG2DC2	CG311	CTL2	CTL2	180	1.738019	3
CTL2	CG311	OG301	OG301	0	-4.33415	1
CTL2	CG311	OG301	OG301	0	0.442003	2
CTL2	CG311	OG301	OG301	0	2.486215	3
OG301	CG311	CTL2	HAL2	0	-0.07253	3
CG311	OG301	OG301	HGP1	0	15.86135	1
CG311	OG301	OG301	HGP1	0	7.457796	2
CG311	OG301	OG301	HGP1	0	1.28234	3
HGA1	CG311	OG301	OG301	0	3.67548	1
HGA1	CG311	OG301	OG301	0	3.084925	3
HGA4	CG2DC2	CG311	OG301	0	-0.81715	3
CG2DC2	CG2DC2	CG311	OG301	180	4.439071	1
CG2DC2	CG2DC2	CG311	OG301	180	0.492857	2
CG2DC2	CG2DC2	CG311	OG301	180	-0.42963	3
CG2DC2	CG311	OG301	OG301	180	15.47172	1
CG2DC2	CG311	OG301	OG301	180	1.109588	2
CG2DC2	CG311	OG301	OG301	180	1.627527	3
HGA1	CG311	CTL2	HAL2	0	1.008433	3
CG2DC2	CG311	CTL2	HAL2	0	0.523045	3
HGA4	CG2DC2	CG311	CTL2	0	0.440828	3
CG2DC2	CG2DC2	CG311	CTL2	180	5.115814	1
CG2DC2	CG2DC2	CG311	CTL2	180	-2.01023	2
CG2DC2	CG2DC2	CG311	CTL2	180	0.128741	3
HGA4	CG2DC2	CG311	HGA1	0	2.073128	3
CG2DC2	CG2DC2	CG311	HGA1	180	3.056732	3
CG311	CG2DC2	CG2DC2	HGA4	180	21.7568	2
CG2DC1	CG2DC2	CG2DC2	CG311	180	2.34304	1
CG2DC1	CG2DC2	CG2DC2	CG311	180	29.288	2
CG2DC2	CG2DC1	CG2DC1	CTL2	180	2.34304	1
CG2DC2	CG2DC1	CG2DC1	CTL2	180	29.288	2
CTL2	CG2DC1	CG2DC1	HGA4	180	21.7568	2
CG2DC1	CG2DC1	CTL2	HAL2	180	1.2552	3
CG2DC1	CG2DC1	CTL2	CTL2	180	3.80744	1
CG2DC1	CG2DC1	CTL2	CTL2	180	0.75312	2
CG2DC1	CG2DC1	CTL2	CTL2	180	0.71128	3
HGA4	CG2DC1	CTL2	CTL2	0	0.50208	3
HGA4	CG2DC1	CTL2	HAL2	0	0	3
CEL1	CEL1	CTL2	CG2DC1	180	4.184	1
CEL1	CEL1	CTL2	CG2DC1	0	0.4184	2
CEL1	CEL1	CTL2	CG2DC1	180	1.2552	3
CEL1	CEL1	CTL2	CG2DC1	0	0.8368	4
CEL1	CTL2	CG2DC1	HGA4	0	0	2
CEL1	CTL2	CG2DC1	HGA4	0	0	3
HEL1	CEL1	CTL2	CG2DC1	0	0	2
HEL1	CEL1	CTL2	CG2DC1	0	0	3
CEL1	CTL2	CG2DC1	CG2DC1	180	4.184	1
CEL1	CTL2	CG2DC1	CG2DC1	0	0.4184	2
CEL1	CTL2	CG2DC1	CG2DC1	180	1.2552	3
CEL1	CTL2	CG2DC1	CG2DC1	0	0.8368	4

Supplementary Table S11. The mass density distribution region and peak site of components for the inner and outer layers of the native and ferroptosis membranes, over the last 400 ns (n = 2).

Membrane components	Inner layer				Outer layer			
	Native membrane		Ferroptosis membrane		Native membrane		Ferroptosis membrane	
	Region range (nm)	Peak site (nm)	Region range (nm)	Peak site (nm)	Region range (nm)	Peak site (nm)	Region range (nm)	Peak site (nm)
Choline head group	-1.8 -3.2	-2.6	-1.6 -3.2	-2.4	1.4 3.0	2.15	1.2 3.0	1.95
Head group of SM	-1.8 -3.2	-2.55	-1.4 -3.2	-2.35	1.6 3.0	2.2	1.2 3.0	2.0
Serine head group	-1.8 -3.4	-2.6	-1.4 -3.4	-2.4	1.8 2.8	2.4	1.4 2.8	2.2
Ethanolamine head group	-1.6 -3.2	-2.4	-1.2 -3.2	-2.2	1.6 2.8	2.15	1.4 2.8	1.95
Phosphate head group	-1.6 -3.2	-2.37	-1.4 -3.2	-2.2	1.4 2.8	2.15	1.2 2.8	2.0
Carbonyl	-1.2 -2.8	-2.0	-1.0 -2.8	-1.9	1.0 2.4	1.6	0.8 2.4	1.5
Polar atoms of SM	-1.4 -2.6	-2.0	-1.2 -2.6	-1.9	1.2 2.4	1.8	1.0 2.4	1.6
sn2- LLA-C9	-0.4 -2.0	-1.0	-0.4 -2.6	-1.4	0.2 1.8	0.95	0.2 2.2	1.2
sn2- LLA-C13	0.0 -1.4	-0.8	0.0 -2.4	-1.0	0.0 1.2	0.6	0.0 2.0	0.9
Sn1- LLA-C13	0.0 -1.0	-0.6	0.0 -1.9	-0.8	0.0 1.2	0.4	0.0 2.0	1.3
sn2-AA-C12	-0.1 -2.0	-1.0	-0.1 -2.6	-1.4	0.1 1.6	0.75	0.1 1.8	1.15
sn2-AA-C15	0.0 -1.8	-0.6	0.0 -2.6	-0.8	0.0 1.4	0.6	0.0 2.0	1.3
Sn1-AA-C15	0.0 -1.6	-0.6	0.0 -2.4	-1.0	0.0 1.4	0.4	0.0 2.0	1.0
sn2-DHA-C14	0.0 -1.8	-0.8	0.0 -2.4	-0.4	0.0 1.4	0.6	0.0 1.6	1.0
sn2-DHA-C17	0.0 -1.4	-0.6	0.0 -2.4	-0.8	0.0 1.2	0.2	0.0 1.8	1.3
sn1-DHA-C17	0.0 -1.2	-0.5	0.0 -2.4	-0.6	0.0 1.0	0.2	0.0 1.8	1.3

Supplementary Table S12. The shifts in the density distribution profiles and peak sites between the ferroptosis and native membranes, over the last 400 ns (n = 2). The “ * ” indicates a shift towards the ferroptosis membrane core, while the “ # ” indicates a shift towards the ferroptosis membrane polar interface.

Membrane components	OXI respect to NOR			
	Inner		Outer	
	Shift in Region (nm)	Shift in Peak site (nm)	Shift in Region (nm)	Shift in Peak site (nm)
Choline head group	0.2^{*}	0.2^{*}	0.2^{*}	0.2^{*}
Head group of SM	0.4^{*}	0.2^{*}	0.4^{*}	0.2^{*}
Serine head group	0.4^{*}	0.2^{*}	0.4^{*}	0.2^{*}
Ethanolamine head group	0.4^{*}	0.2^{*}	0.2^{*}	0.2^{*}
Phosphate head group	0.2^{*}	0.17^{*}	0.2^{*}	0.15^{*}
Carbonyl	0.2^{*}	0.1^{*}	0.2^{*}	0.1^{*}
Polar atoms of SM	0.2^{*}	0.1^{*}	0.2^{*}	0.2^{*}
sn2-C9 (LLA)	$0.6^{\#}$	$0.4^{\#}$	$0.4^{\#}$	$0.25^{\#}$
sn2-C13 (LLA)	$1.0^{\#}$	$0.2^{\#}$	$0.8^{\#}$	$0.3^{\#}$
Sn1-C13 (LLA)	$0.9^{\#}$	$0.2^{\#}$	$0.8^{\#}$	$0.9^{\#}$
sn2-C12 (AA)	$0.6^{\#}$	$0.4^{\#}$	$0.2^{\#}$	$0.4^{\#}$
sn2-C15 (AA)	$0.8^{\#}$	$0.2^{\#}$	$0.6^{\#}$	$0.7^{\#}$
Sn1-C15 (AA)	$0.8^{\#}$	$0.4^{\#}$	$0.6^{\#}$	$0.6^{\#}$
sn2-C14 (DHA)	$0.6^{\#}$	0.4^{*}	$0.2^{\#}$	$0.4^{\#}$
sn2-C17 (DHA)	$1.0^{\#}$	$0.2^{\#}$	$0.6^{\#}$	$1.1^{\#}$
sn1-C17 (DHA)	$1.2^{\#}$	$0.1^{\#}$	$0.8^{\#}$	$1.1^{\#}$

Supplementary Table S13. The thickness and area per lipid of the native and ferroptosis membranes (over the last 400 ns (n = 2)) were compared using both human erythrocyte membrane experimental data and simple bilayer simulation data.

	**Membrane thickness (nm)**	**APL ($nm^{2)}$**
The inner layer of the native membrane	**2.37**	**0.45**
The outer layer of the native membrane	**2.15**	**0.50**
The inner layer of the ferroptosis membrane	**2.2**	**0.51**
The outer layer of the ferroptosis membrane	**2.0**	**0.56**
Lipid domains in liquid-ordered aphase[a]	**4.60**	**0.38**
Lipid domains in liquid disordered phase[a]	**4.10**	**0.50**
POPC bilayers[b] (pure)	**3.94**	**0.63**
POPC bilayers[b] (15% oxi)	**3.64**	**0.66**
PLPC[c] (pure)	**3.62**	**0.65**
PLPC[c]-9-oxi (25% oxi)	**3.60**	**0.68**
PLPC[c]-9-oxi (50% oxi)	**3.47**	**0.70**
PLPC[c]-13-oxi (25% oxi)	**3.54**	**0.69**
PLPC[c]-13-oxi (50% oxi)	**3.33**	**0.71**

[a] The structure of the lipid domains in the erythrocyte membrane, where the lipid chains are in either an all-trans configuration (ordered) or a bent configuration (disordered), was determined using high-resolution X-ray diffraction [1].
[b] The all-atom simulation [2].
[c] The all-atom simulation [3].

Supplementary Table S14. The average tilt and inner angles of the phospholipids containing LA tails (in the native membrane) and hydroperoxidized LA tails (in the ferroptosis membrane) were calculated over the last 400 ns (n = 2). The angles were defined in Figure 7 as follows: the “ * ” refers to the average values (‘A’), and “ # ” refers to the standard deviation (‘d’).

Phospholipids	Inner layer									Outer layer								
	α1°	α2°	β°	γ1°	γ2_1°	γ2_2°	δ1°	δ2_1°	δ2_2°	α1°	α2°	β°	γ1°	γ2_1°	γ2_2°	δ1°	δ2_1°	δ2_2°
	A* d#	A d	A d	A d	A d	A d	A d	A d	A d	A d	A d	A d	A d	A d	A d	A d	A d	A d
PLPE	18 4	13 7	21 7	-	148 17	137 17	-	14 5	13 4	-	-	-	-	-	-	-	-	-
PLPE9	20 8	36 17	39 11	-	114 31	-	-	38 19	-	-	-	-	-	-	-	-	-	-
PLPE13	21 13	31 16	40 13	-	-	96 34	-	-	33 10	-	-	-	-	-	-	-	-	-
SLPE	17 5	14 4	20 6	-	157 13	150 11	-	15 5	15 3	17 5	16 5	21 5	-	144 20	132 19	-	19 7	17 5
SLPE9	25 7	36 14	32 18	-	126 35	-	-	28 17	-	32 13	29 11	46 23	-	114 36	-	-	40 15	-
SLPE13	19 9	35 22	39 16	-	-	94 36	-	-	45 19	23 10	38 12	38 17	-	-	114 38	-	-	38 15
DLPE	23 6	15 9	23 10	137 39	152 17	142 25	25 6	16 9	14 6	-	-	-	-	-	-	-	-	-
DLPE9,13	24 5	37 13	30 11	124 33	112 32	-	27 7	47 23	-	-	-	-	-	-	-	-	-	-
PLPC	18 4	12 7	24 8	-	154 10	146 14	-	17 6	16 5	22 3	21 3	25 3		138 11	126 14		20 5	18 4
PLPC9	19 9	29 13	25 10	-	129 23	-	-	25 18	-	26 8	32 9	35 8	-	110 24	-	-	51 13	-
PLPC13	20 8	38 19	33 13	-	-	109 41	-	-	58 18	25 7	37 9	45 14	-	-	112 30	-	-	45 14
SLPC	18 6	13 6	20 6	-	142 19	135 23	-	17 5	13 6	20 4	17 6	24 5		140 17	126 14		17 5	15 5
SLPC9	24 12	17 8	25 12	-	133 27	-	-	20 10		26 8	28 10	37 11	-	114 30	-	-	53 11	-
SLPC13	17 9	21 13	31 10	-	-	127 43	-	-	28 13	26 6	43 11	52 15	-	-	138 17	-	-	58 13
OLPC	17 4	14 9	19 7	-	149 17	140 22	-	15 6	14 6	20 6	18 7	25 4		134 21	122 23		20 6	16 6
OLPC9	21 11	40 23	47 24	-	106 37	-	-	41 31	-	33 12	39 16	50 17	-	100 24	-	-	66 18	-
OLPC13	19 13	31 12	41 15	-	-	130 29	-	-	39 15	27 9	31 14	41 20	-	-	110 40			34 19
DLPC	22 7	11 7	19 6	142 25	156 14	148 17	26 5	18 8	15 5	25 7	24 7	34 10	116 17	124 19	120 14	28 8	20 7	27 6
DLPC9,13	25 14	28 12	37 18	136 34	128 30	-	30 18	28 13	-	30 10	27 6	39 9	103 27	104 16	-	52 21	60 16	-
SLPS	20 6	12 9	22 7		147 13	140 18		18 8	14 7	-	-	-	-	-	-	-	-	-
SLPS9	22 10	24 11	32 9	-	140 46	-	-	26 12	-	-	-	-	-	-	-	-	-	-
SLPS13	17 11	36 13	34 14	-	-	126 34	-	-	54 13	-	-	-	-	-	-	-	-	-
LNSM	-	-	-	-	-	-	-	-	-	22 7	25 6	33 12	-	140 14	133 25	-	25 7	22 9
LNSM9	-	-	-	-	-	-	-	-	-	24 12	33 15	36 15	-	112 33	-	-	58 21	-
LNSM13	-	-	-	-	-	-	-	-	-	25 11	29 10	38 10	-	-	102 37	-	-	45 12

Supplementary Table S15. The average tilt and inner angles of the phospholipids containing AA tails (in the native membrane) and hydroperoxidized AA tails (in the ferroptosis membrane) were calculated over the last 400 ns (n = 2). The angles were defined in Figure 7 as follows: the " * " refers to the average values ('A'), and " # " refers to the standard deviation ('d').

	Inner layer									Outer layer								
Phospholipids	α1°	α2°	β°	γ1°	γ2_1°	γ2_2°	δ1°	δ2_1°	δ2_2°	α1°	α2°	β°	γ1°	γ2_1°	γ2_2°	δ1°	δ2_1°	δ2_2°
	A* d#	A d	A d	A d	A d	A d	A d	A d	A d	A d	A d	A d	A d	A d	A d	A d	A d	A d
SAPE	18 2	18 2	20 3	-	144 8	139 8	-	20 4	16 3	21 6	36 9	39 12	-	120 25	118 29	-	34 9	35 8
SAPE12	24 4	25 5	32 7	-	132 10	-	-	34 8	-	26 14	39 9	42 15	-	117 20	-	-	64 20	-
SAPE15	23 5	26 6	33 9	-	-	124 10	-	-	30 5	24 11	37 17	44 20	-	-	117 32	-	-	49 22
DAPE	16 4	19 7	24 7	135 19	137 20	133 21	21 5	20 5	22 7	21 11	31 13	36 16	127 39	124 31	114 39	20 11	34 10	37 11
DAPE12,15	23 4	24 10	31 8	127 18	130 15	-	28 9	36 9	-	29 15	33 14	40 18	100 19	117 24	-	48 21	54 19	-
SAPC	17 3	15 4	20 5	-	149 11	140 13	-	15 5	19 5	23 6	30 6	38 10	-	121 16	126 16	-	31 8	30 9
SAPC12	21 7	31 13	29 15	-	123 23	-	-	29 20	-	26 9	31 11	42 11	-	118 17	-	-	48 17	-
SAPC15	20 7	28 20	37 20	-	-	126 39	-	-	37 19	25 7	33 10	39 10	-	-	120 20	-	-	45 18
DAPC	-	-	-	-	-	-	-	-	-	25 8	37 17	35 15	123 34	124 35	114 38	28 12	29 12	31 15
DAPC12,15	-	-	-	-	-	-	-	-	-	31 11	39 15	41 17	81 14	120 29	-	49 20	55 21	
SAPS	18 2	18 2	20 2	-	146 6	133 9	-	19 2	16 2	17 5	34 10	33 12	-	127 22	120 23	-	35 7	34 8
SAPS12	22 5	23 5	30 7	-	136 10	-	-	29 7	-	29 10	36 12	44 21	-	118 30	-	-	58 20	-
SAPS15	23 4	26 7	35 7	-	-	123 14	-	-	31 4	27 9	36 16	39 20	-	-	114 37	-	-	57 25
DAPS	16 5	17 5	20 5	139 20	150 15	141 21	19 6	15 7	19 7	-	-	-	-	-	-	-	-	-
DAPS12,15	25 11	30 14	43 19	130 30	120 21	-	25 11	44 22	-	-	-	-	-	-	-	-	-	-

Supplementary Table S16. The average tilt and inner angles of the phospholipids containing DHA tail (in the native membrane) and hydroperoxidized DHA tails (in the ferroptosis membrane) were calculated over the last 400 ns (n = 2). The angles were defined in Figure 7 as follows: the symbol “ * ” refers to the average values (‘A’), and “ # ” refers to the standard deviation (‘d’).

Phospholipids	Inner layer									Outer layer								
	α1°	α2°	β°	γ1°	γ2_1°	γ2_2°	δ1°	δ2_1°	δ2_2°	α1°	α2°	β°	γ1°	γ2_1°	γ2_2°	δ1°	δ2_1°	δ2_2°
	A* d#	A d	A d	A d	A d	A d	A d	A d	A d	A d	A d	A d	A d	A d	A d	A d	A d	A d
SDPE	12 3	17 5	20 4	-	140 10	131 12		20 4	17 3	18 5	24 8	25 8	-	119 23	116 25	-	20 7	21 6
SDPE14	25 7	30 10	32 9	-	132 20	-	-	29 10	-	29 12	31 15	30 14	-	116 28	-	-	45 17	-
SDPE17	35 8	28 5	31 6	-	-	118 16	-	-	36 6	29 8	35 14	30 18	-	-	120 28	-	-	45 14
ODPE	15 4	19 5	25 8	-	135 16	129 17		20 6	15 5	-	-	-	-	-	-	-	-	-
ODPE14	33 10	35 12	30 11	-	124 34	-	-	27 12	-	-	-	-	-	-	-	-	-	-
ODPE17	29 15	30 16	41 17	-	-	113 36	-	-	34 16	-	-	-	-	-	-	-	-	-
DDPE	14 4	17 5	21 5	135 14	138 11	132 15	11 5	19 6	17 4	34 13	25 16	35 20	121 35	113 29	117 35	20 14	25 15	29 12
DDPE14,17	30 8	35 11	33 9	122 20	118 17	-	39 9	30 10	-	40 20	26 11	40 19	77 30	110 25	-	45 20	35 15	-
SDPC	-	-	-	-	-	-	-	-	-	20 7	27 10	26 10	-	122 20	126 23	-	27 9	26 8
SDPC14	-	-	-	-	-	-	-	-	-	30 11	40 15	30 16	-	114 24	-	-	60 21	-
SDPC17	-	-	-	-	-	-	-	-	-	41 18	35 17	43 18	-	-	106 29	-	-	65 30
SDPS	13 2	19 3	20 2	-	140 7	134 8		20 3	17 3	-	-	-	-	-	-	-	-	-
SDPS14	26 7	31 9	32 5	-	125 12	-	-	30 8	-	-	-	-	-	-	-	-	-	-
SDPS17	33 8	47 8	35 7	-	-	110 14	-	-	44 12	-	-	-	-	-	-	-	-	-
DDPS	17 10	17 9	26 12	121 32	131 31	125 34	13 7	19 10	22 8	-	-	-	-	-	-	-	-	-
DDPS14,17	40 14	29 10	42 16	114 35	125 17	-	44 14	43 15		-	-	-	-	-	-	-	-	-
DSM	-	-	-	-	-	-	-	-	-	20 9	32 13	40 15	-	126 30	121 29	-	30 12	25 11
DSM14	-	-	-	-	-	-	-	-	-	32 14	41 14	45 21	-	118 20	-	-	62 22	-
DSM17	-	-	-	-	-	-	-	-	-	33 13	57 15	44 16	-	-	103 30	-	-	69 23

Supplementary Table S17. The average tilt and inner angles of the phospholipids containing saturated tails in both the native and ferroptosis membranes were calculated over the last 400 ns (n = 2). The angles were defined in Figure 7 as follows: the symbol " * " refers to the average values ('A'), and " # " refers to the standard deviation ('d').

Phospholipids	Inner layer						Outer layer					
	α1°		α2°		β°		α1°		α2°		β°	
	A*	d#	A	d	A	d	A	d	A	d	A	d
POPE	17	2	16	3	27	5	20	5	26	4	28	6
POPE*	19	7	26	5	29	7	22	7	22	6	29	7
DPPE	16	5	15	3	21	3	-		-		-	
DPPE*	20	9	26	9	23	6	-		-		-	
POPC	16	4	17	5	31	5	22	3	24	3	29	5
POPC*	21	8	27	6	33	9	24	5	28	4	31	6
DPPC	15	3	16	5	25	5	20	4	23	5	28	4
DPPC*	20	6	24	7	27	7	23	4	27	6	32	8
SOPS	14	3	17	4	26	4	-		-		-	
SOPS*	18	6	27	6	28	6	-		-		-	
DSPS	17	4	15	3	22	4	23	7	21	6	32	8
DSPS*	20	7	23	8	24	8	26	9	24	10	35	11
PSM	14	5	17	5	25	5	18	3	23	4	26	5
PSM*	19	9	25	10	26	7	19	4	25	5	28	4
SSM	15	6	19	8	27	6	20	4	24	6	30	7
SSM*	19	10	28	12	28	14	22	6	26	5	31	6
BSM	-		-		-		16	4	23	5	29	8
BSM*	-		-		-		18	7	27	7	30	8
LSM	14	4	19	8	23	9	18	2	26	4	27	5
LSM*	18	7	24	10	25	10	19	5	30	4	28	6
NSM	16	5	17	7	23	5	19	2	25	3	31	6
NSM*	20	8	23	8	25	9	21	2	29	3	32	5

Supplementary Table S18. The average long-time lateral diffusion coefficients for all lipids in each monolayer of both the native and ferroptosis membranes were calculated over the last 400 ns (n = 2). The terms “e.e”, “SD”, and “PL” refer to the error estimate, standard deviation, and phospholipids, respectively.

Phospholipids	Native membrane						Ferroptosis membrane					
	D ($\mu m^2.s^{-1}$) Inner layer			D ($\mu m^2.s^{-1}$) Outer layer			D ($\mu m^2.s^{-1}$) Inner layer			D ($\mu m^2.s^{-1}$) Outer layer		
	Avg	e.e	SD	Avg	e.e	SD	Avg	e.e	SD	Avg	e.e	SD
DPPE	2.5	0.2	0.5		-		1.0	1.0	3.2		-	
POPE	3.1	0.3	2.8	4.9	0.3	1.9	2.5	0.2	2.2	2.9	0.8	1.6
PLPE	2.0	0.5	1.2		-			-			-	
PLPE9		-			-		3.0	0.3	0.0		-	
PLPE13		-			-		1.3	0.1	0.0		-	
SLPE	3.5	0.7	1.4	1.9	0.2	3.6		-			-	
SLPE9		-			-		2.6	0.2	0.0	0.9	0.0	0.0
SLPE13		-			-		1.6	0.1	0.0	1.2	0.0	0.0
DLPE	3.0	0.1	0.0		-			-			-	
DLPE9,13		-			-		4.0	0.1	0.0			
SAPE	2.1	0.3	1.8	3.3	0.1	0.4		-			-	
SAPE12		-			-		1.5	0.2	2.2	1.2	0.0	0.0
SAPE15		-			-		1.6	0.3	1.7	2.0	0.0	0.0
DAPE	4.4	0.4	1.6	4.0	0.2	0.0		-			-	
DAPE12,15		-			-		3.5	0.4	2.0	1.9	0.0	0.0
SDPE	3.7	0.5	4.2	2.5	0.3	4.3		-			-	
SDPE14		-			-		1.2	0.5	1.9	1.5	0.0	0.0
SDPE17		-			-		1.8	0.3	0.5	2.0	0.0	0.0
ODPE	3.3	0.1	0.2		-			-			-	
ODPE14		-			-		1.0	0.1	0.0		-	
ODPE17		-			-		0.5	0.1	0.0		-	
DDPE	1.0	0.4	1.6	3.6	0.1	0.0		-			-	
DDPE14,17		-			-		0.7	0.2	0.7	1.4	0.0	0.0
POPC	2.5	0.6	0.2	4.2	0.3	2.2	2.0	0.3	1.5	3.4	0.9	3.2
DPPC	3.0	0.3	1.0	3.8	0.4	4.2	1.0	0.3	0.6	4.0	0.7	3.8
PLPC	0.8	0.2	0.2	5.5	0.5	2.5		-			-	
PLPC9		-			-		1.0	0.2	0.0	4.5	0.8	1.4
PLPC13		-			-		2.0	0.3	0.0	1.5	0.2	0.3
SLPC	2.4	0.4	1.3	3.0	0.3	1.6		-			-	
SLPC9		-			-		4.3	0.1	0.0	2.5	1.2	1.8
SLPC13		-			-		4.5	0.1	0.0	3.8	0.8	2.8
OLPC	3.0	0.3	2.9	4.0	0.4	4.3		-			-	
OLPC9		-			-		0.5	0.2	0.0	3.4	0.0	0.0
OLPC13		-			-		1.0	0.1	0.0	3.0	0.0	0.0
DLPC	3.7	0.2	0.0	4.7	0.3	1.5		-			-	
DLPC9,13		-			-		0.5	0.0	0.0	1.4	0.3	0.5
SAPC	1.2	0.1	0.0	3.6	0.2	1.2		-			-	
SAPC12		-			-		1.4	0.1	0.0	1.5	0.6	0.9
SAPC15		-			-		2.2	0.0	0.0	0.3	0.2	0.3
DAPC		-		3.0	0.1	0.0		-			-	
DAPC12,15		-			-			-		0.5	0.0	0.0
SDPC		-		3.9	0.2	0.4		-			-	
SDPC14		-			-			-		0.9	0.0	0.0
SDPC17		-			-					2.7	0.0	0.0
DSPS	3.0	0.3	2.6	4.0	0.1	0.0	2.6	0.3	0.5	1.8	0.0	0.0
SOPS	1.0	0.4	1.8		-		1.8	0.2	2.2		-	
SLPS	3.2	0.2	0.1		-			-			-	
SLPS9		-			-		2.0	0.0	0.0		-	
SLPS13		-			-		3.0	0.1	0.0		-	
SAPS	1.5	0.5	2.8	3.0	0.2	0.5		-			-	
SAPS12		-			-		1.6	0.2	1.7	4.2	0.0	0.0
SAPS15		-			-		2.5	0.3	2.3	2.4	0.0	0.0
DAPS	1.2	0.1	0.0		-			-			-	
DAPS12,15		-			-		0.9	0.0	0.0		-	
SDPS	2.1	0.2	1.5		-			-			-	
SDPS14		-			-		1.0	0.3	4.2		-	
SDPS17		-			-		2.0	0.2	1.7		-	
DDPS	2.1	0.3	0.0		-			-			-	
DDPS14,17		-			-		1.0	0.0	0.0		-	
PSM	2.5	0.2	0.5	3.6	0.6	1.3	1.9	0.1	0.2	2.3	1.0	1.7
SSM	2.2	0.1	0.0	4.0	0.4	1.5	1.9	0.1	0.0	2.2	0.4	1.5

LNSM		-		3.3	0.3	1.4		-			-	
LNSM9		-			-			-		0.3	0.0	0.0
LNSM13		-			-			-		0.5	0.0	0.0
LSM	0.5	0.3	0.0	3.9	0.4	2.3	0.4	0.0	0.0	2.2	0.5	1.4
BSM		-		3.2	0.5	2.0		-		2.4	0.5	0.8
DSM	3.1	0.2	0.0	1.6	0.1	0.0		-			-	
DSM14		-			-		2.0	0.1	0.0	1.0	0.0	0.0
DSM17		-			-		1.8	0.0	0.0	0.2	0.0	0.0
NSM	3.1	0.4	1.0	3.5	0.4	1.4	1.0	0.2	0.9	2.5	0.7	1.4
Cholesterol	2.4	0.7	2.1	5.0	1.3	3.8	2.4	0.3	2.3	3.1	0.3	2.4
Total PLs	2.5	-	1.7	3.6	-	1.1	1.9	-	2.0	1.9	-	1.2

References

1. Himbert, S., et al., *The molecular structure of human red blood cell membranes from highly oriented, solid supported multi-lamellar membranes.* Scientific reports, 2017. **7**(1): p. 39661.
2. Yang, H., et al., *Effects of low-level lipid peroxidation on the permeability of nitroaromatic molecules across a membrane: a computational study.* ACS omega, 2020. **5**(10): p. 4798-4806.
3. Wong-Ekkabut, J., et al., *Effect of lipid peroxidation on the properties of lipid bilayers: a molecular dynamics study.* Biophysical journal, 2007. **93**(12): p. 4225-4236.
